# Perception of a divergent family of phytocytokines by the Arabidopsis receptor kinase MIK2

**DOI:** 10.1038/s41467-021-20932-y

**Published:** 2021-01-29

**Authors:** Jack Rhodes, Huanjie Yang, Steven Moussu, Freddy Boutrot, Julia Santiago, Cyril Zipfel

**Affiliations:** 1 The Sainsbury Laboratory, University of East Anglia, Norwich Research Park, Norwich, UK; 2 Institute of Plant and Microbial Biology, Zurich-Basel Plant Science Center, University of Zurich, Zurich, Switzerland; 3 The Plant Signaling Mechanisms Laboratory, Department of Plant Molecular Biology, University of Lausanne, Lausanne, Switzerland; 4 Present Address: Anova-Plus, Évry, Évry-Courcouronnes, France

**Keywords:** Pattern recognition receptors, Pattern recognition receptors in plants, Plant signalling

## Abstract

Plant genomes encode hundreds of receptor kinases and peptides, but the number of known plant receptor-ligand pairs is limited. We report that the *Arabidopsis* leucine-rich repeat receptor kinase LRR-RK MALE DISCOVERER 1-INTERACTING RECEPTOR LIKE KINASE 2 (MIK2) is the receptor for the SERINE RICH ENDOGENOUS PEPTIDE (SCOOP) phytocytokines. MIK2 is necessary and sufficient for immune responses triggered by multiple SCOOP peptides, suggesting that MIK2 is the receptor for this divergent family of peptides. Accordingly, the SCOOP12 peptide directly binds MIK2 and triggers complex formation between MIK2 and the BRASSINOSTEROID INSENSITIVE 1-ASSOCIATED KINASE 1 (BAK1) co-receptor. MIK2 is required for resistance to the important root pathogen *Fusarium oxysporum*. Notably, we reveal that *Fusarium* proteomes encode SCOOP-like sequences, and corresponding synthetic peptides induce MIK2-dependent immune responses. These results suggest that MIK2 may recognise *Fusarium*-derived SCOOP-like sequences to induce immunity against *Fusarium*. The definition of SCOOPs as MIK2 ligands will help to unravel the multiple roles played by MIK2 during plant growth, development and stress responses.

## Abstract (web summary)

Secreted peptides and cell-surface localized receptor kinases allow plants to modify growth and development according to external cues. Here, Rhodes et al. show that the MIK2 receptor perceives the SERINE RICH ENDOGENOUS PEPTIDE (SCOOP) family of phytocytokines and is capable of recognising *Fusarium*-derived SCOOP-like peptides.

## Introduction

Plants employ cell-surface localized receptors, such as receptor kinases (RKs), to sense their extracellular environment and coordinate their growth and development in response to endogenous and exogenous cues^1^. Secreted plant peptides—encoded by hundreds of genes in plant genomes—have recently emerged as playing a major role in this autocrine and paracrine communication and are proposed as a new class of plant hormones^2^. The number of known receptors for such peptides however remains scarce.

The leucine-rich repeat receptor kinase (LRR-RK) MALE DISCOVERER 1-INTERACTING RECEPTOR-LIKE KINASE 2 (MIK2) was initially identified for its proposed role in pollen tube guidance mediated by the cysteine-rich AtLURE1 peptides. In this context, it was shown to interact with the LRR-RK MALE DISCOVERER 1 (MDIS1), and was therefore proposed to be part of a receptor complex for AtLURE1 peptides^3,4^. At the same time, another set of LRR-RKs, POLLEN-SPECIFIC RECEPTOR-LIKE KINASE 6 (PRK6) and related proteins, were shown to be involved in AtLURE1 perception^5^. Structural and biochemical work then showed that AtLURE1 peptides bind to PRK6, but not to MIK2^6^, and the genetic role of PRK6 in AtLURE1 perception was recently corroborated^7^. These findings questioned whether AtLURE1s are ligands for a potential MDIS1–MIK2 complex. Accordingly, it was later revealed that MIK2 (also named LEUCINE-RICH REPEAT KINASE FAMILY PROTEIN INDUCED BY SALT STRESS, or LRR-KISS), but not MDIS1, is involved in other physiological processes, such as stress responses upon cell wall damage, salt tolerance, root growth, and resistance to the important vascular fungal pathogen *Fusarium oxysporum*^8–11^. None of these processes are known to involve AtLURE1 peptides, and thus the identity and origin of the MIK2 ligand(s) remain unknown.

Here, we identify MIK2 as the receptor of the SCOOP family of phytocytokines. SCOOP12 binds directly to MIK2 to induce MIK2–BAK1 complex formation and activation of downstream signaling. All tested SCOOP peptides similarly induce MIK2-dependent responses and MIK2–BAK1 complex formation. Moreover, we identify SCOOP-like sequences within *Fusarium* proteomes, whose corresponding synthetic peptides induce MIK2-dependent responses and MIK2–BAK1 association, suggesting MIK2 may directly perceive *Fusarium*.

## Results and discussion

**Fig. 1:**
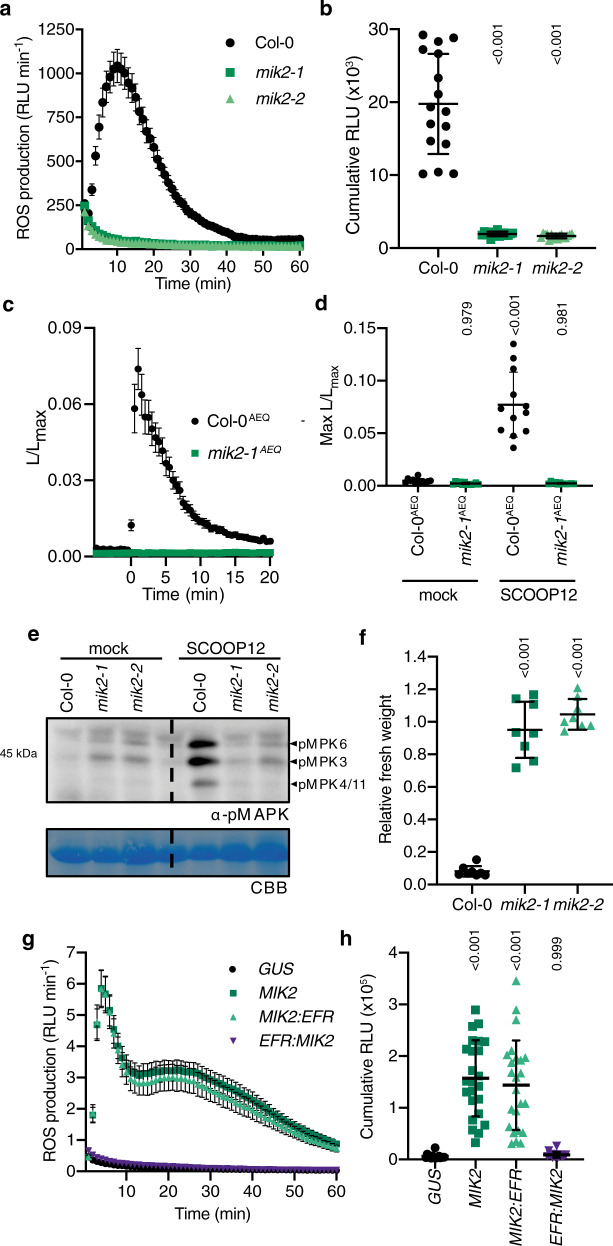
MIK2 is required and sufficient for SCOOP12-induced responses. **a**, **b** ROS production in leaf disks collected from 4-week-old *A. thaliana* plants induced by 1 μM SCOOP12 application (*n* = 16 leaf disks). **a** Points represent mean; error bars represent S.E.M. **b** Integrated ROS production over 40 min. Line represents mean; error bars represent S.D. **c**, **d** The *mik2-1* mutation was crossed into the *pUBQ10::AEQUORIN* background, and calcium levels were measured in seedlings relative to maximum upon discharge following the addition of 100 nM SCOOP12 (*n* = 12 seedlings). **c** Points represent mean; error bars represent S.E.M. **d** Maximum relative SCOOP12-induced calcium levels ±S.D. **e** Western blot using α-p42/p44-ERK recognizing phosphorylated MPK6, MPK3, and MPK4/11 in seedlings treated with 100 nM SCOOP12 or mock for 15 min. Membranes were stained with CBB, as a loading control. **f** Fresh weight of 14-day-old seedlings grown in the presence of 1 μM SCOOP12 for 10 days relative to mock (*n* = 8 seedlings). A line represents mean; error bars represent S.D. **g**, **h**) ROS production in leaf disks of *N. benthamiana* transiently expressing the defined constructs induced by 1 μM SCOOP12 application (*n* = 24 leaf disks). **g** Points represent mean; error bars represent S.E.M. **h** Integrated ROS production over 60 min. A line represents mean; error bars represent S.D. *P*-values indicate significance relative to the WT control (or GUS transformed control) in a Tukey’s multiple comparisons test following one-way ANOVA. All experiments were repeated and analyzed three times with similar results. ROS reactive oxygen species, CBB Coomassie brilliant blue.

In an effort to understand how MIK2 regulates stress responses, we tested whether *Arabidopsis thaliana* (hereafter, *Arabidopsis*) *mik2* mutant plants are affected in the production of reactive oxygen species (ROS) upon treatment with different immune elicitors. A clear defect in apoplastic ROS production was observed in *mik2* plants for some, but not all elicitors tested based on elicitor origin (Supplementary Fig. 1). As a way to test the generality of these observations, other microbial and plant elicitors were tested, including the recently identified SERINE RICH ENDOGENOUS PEPTIDE 12 (SCOOP12) peptide^12^ that is proposed to function as a phytocytokine (a secreted peptide that regulates plant immune responses by analogy to cytokines in metazoans^13^). Surprisingly, unlike what was observed with flg22 or Pep1 (Supplementary Fig. 1), *mik2* plants were insensitive to SCOOP12 treatment for all cellular responses measured (Fig. 1a–f and Supplementary Fig. 2a–d), i.e. ROS production increase in cytosolic Ca^2+^ concentration, mitogen-activated protein kinase phosphorylation, and seedling growth inhibition—all of which being hallmarks of immune signaling^14^. Importantly, the loss of *MIK2* could be complemented with the expression of *MIK2* under its native promoter (Supplementary Fig. 2e, f). That *mik2* plants are blind to SCOOP12 suggests that MIK2 is the receptor for SCOOP12.

Plant cell-surface immune receptors (called pattern recognition receptors) are either RKs or receptor proteins (RPs), the latter lacking any obvious intracellular signaling domain^15,16^. The majority of known receptors for plant-derived or exogenous peptides are LRR-RKs or LRR-RPs that form ligand-induced complexes with LRR-RK co-receptors belonging to the SOMATIC EMBRYOGENESIS RECEPTOR KINASE (SERK) family^1,17^. Notably, SCOOP12-induced seedling growth inhibition was impaired in a *bak1* (BRASSINOSTEROID INSENSITIVE 1-ASSOCIATED KINASE 1, BAK1 corresponds to SERK3) null mutant^12^. Similarly, we found that SCOOP12-induced ROS production is impaired in *bak1-5* (Supplementary Fig. 3a, b), a *bak1* allele that has a dominant-negative effect on other SERKs^18–20^. Furthermore, SCOOP12 treatment-induced BAK1 phosphorylation on serine 612 (Ser612) (Supplementary Fig. 3c)—a post-translational modification required for immune signaling^20^. By comparison, SCOOP12-induced ROS production was not affected in *sobir1-13* (Supplementary Fig. 3a, b), a null allele of the adapter LRR-RK SUPPRESSOR OF BAK1-INTERACTING RECEPTOR-LIKE KINASE 1 (SOBIR1)^21^, required by LRR-RPs to form active signaling complexes with SERK co-receptors^15,16^. Together, these data suggest that the SCOOP12 receptor is an LRR-RK. Consistently, we also found that optimal SCOOP12-induced ROS production occurs via components involved downstream of other LRR-RK-type PRRs^22–25^ (Supplementary Fig. 3d, e).

To further test if MIK2 is the SCOOP12 receptor, we transiently expressed MIK2 or a chimera between the MIK2 ectodomain and the intracellular domain of EF-TU RECEPTOR (EFR)^26^ (MIK2–EFR) in *Nicotiana benthamiana*. This plant species is insensitive to SCOOP12^12^ and its genome lacks any obvious MIK2 ortholog (Supplementary Fig. 4a–c). However, upon expression of MIK2 or MIK2–EFR, we could measure SCOOP12-induced ROS production in *N. benthamiana*, which was not observed when expressing a transformation control (β-glucuronidase, GUS) or when expressing the reverse chimera EFR-MIK2 that is functional in elf18 perception (Fig. 1g, h and Supplementary Fig. 4d–f). This data demonstrates that MIK2 is sufficient to confer SCOOP12 responsiveness, and indicates that the MIK2 ectodomain is involved in this recognition.

**Fig. 2:**
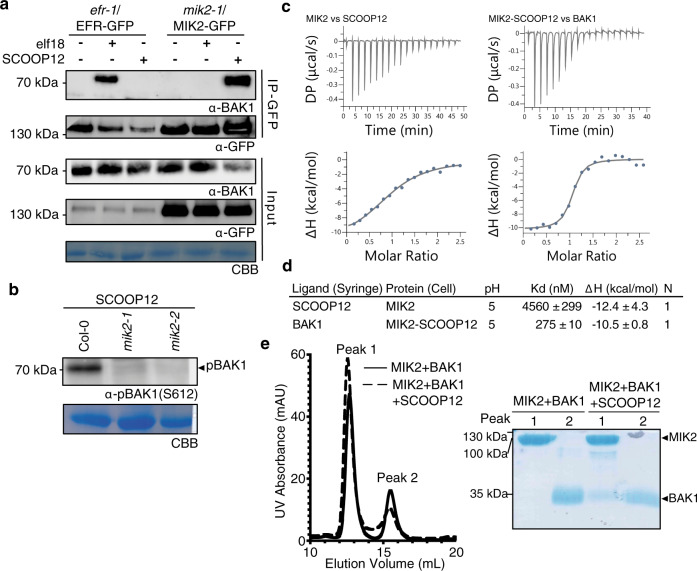
SCOOP12 induces MIK2–BAK1 complex formation. **a** Co-immunoprecipitation of BAK1 with MIK2–GFP from *mik2-1*/MIK2-GFP and EFR-GFP from *efr-1*/EFR-GFP seedlings treated with 1 μM elf18, 1 μM SCOOP12, or water for 10 min. Western blots were probed with antibodies α-GFP and α-BAK1. **b** Western blot using α-pBAK1(Ser612) of seedlings after 15 min treatment with 100 nM SCOOP12. **a**, **b** were repeated three times with similar results. **c** ITC experiments of MIK2 vs SCOOP12, and MIK2–SCOOP12 complex vs BAK1. Representative raw thermogram plots of ITC experiments. **d** ITC table summarizes of MIK2 vs SCOOP12, and MIK2–SCOOP12 vs BAK1. The binding affinities between MIK2 and SCOOP12, and MIK2–SCOOP12 and BAK1, are reported as K_*d*_, (dissociation constant, in nanomoles). The *N* indicates the reaction stoichiometry (*N* = 1 for a 1:1 interaction). ΔH indicates the enthalpy variation. Values indicated in the table are means ± SD of independent experiments (*n* = 3). Corresponding ITC runs are reported in Supplementary Figure 6. **e** Analytical SEC (left panel) of MIK2–BAK1 complex in the presence and absence of SCOOP12. An SDS-PAGE of the peak fractions is shown alongside (right panel). ITC isothermal titration calorimetry, SEC size-exclusion chromatography.

The current paradigm is that ligand-binding LRR-RKs undergo ligand-induced heterodimerization with a shape complementary co-receptor of the SERK family (or a related member of LRR-RK subfamily II) in order to activate signaling^17,27,28^. Having shown that SCOOP12-induced responses are BAK1-dependent (Supplementary Fig. 3a–c and ref. ^12^), we tested whether SCOOP12 could induce MIK2–BAK1 complex formation. Stably expressed MIK2 fused to fluorescent epitope tags (MIK2–FP) co-immunoprecipitated endogenous BAK1 specifically upon treatment with SCOOP12 (Fig. 2a and Supplementary Fig. 5). In contrast, BAK1 co-immunoprecipitated with EFR-GFP upon elf18 but not SCOOP12 treatment in *Arabidopsis* (Fig. 2a). Consistent with this specific ligand-induced heteromerization, SCOOP12-induced BAK1-S612 phosphorylation was abolished in *mik2* mutant plants (Fig. 2b). Finally, we tested if the MIK2 ectodomain (MIK2^ECD^) is sufficient for SCOOP12 binding in vitro. For this, we heterologously expressed MIK2^ECD^ (residues 1–709) in insect cells, and performed binding assays using isothermal titration calorimetry. MIK2^ECD^ directly senses SCOOP12 with a binding affinity of ~4.6 µM. We next quantified the affinity of the co-receptor to the MIK2–SCOOP12 complex. In the presence of SCOOP12, BAK1 strongly binds MIK2 with a dissociation constant in the mid-nanomolar range (~275 nM), which is comparable to its bioactivity (Fig. 2c, d and Supplementary Figs. 6, 7a). This suggests that SCOOP12 itself promotes receptor-co-receptor association, as previously reported for other ligand-LRR-RK pairs^17,29^. This result is in good agreement with the formation of a stable, SCOOP12-dependent MIK2–BAK1 complex in size-exclusion chromatography experiments (Fig. 2e). Together with the SERK-dependency of SCOOP12-induced responses (Supplementary Fig. 3a–c and ref. ^12^) and SCOOP12-induced MIK2–BAK1/SERK association in planta (Fig. 2a and Supplementary Fig. 5), these in vitro data demonstrate that MIK2 is sufficient for SCOOP12 perception, and thus that MIK2 is the SCOOP12 receptor.

*PROSCOOP12* is part of a 14-member family in *Arabidopsis* defined by the presence of a signal peptide, a pro-peptide region, a putative protease cleavage site, and a predicted mature peptide encompassing a serine- and glycine-rich 13-amino-acid epitope that was shown, at least for SCOOP12, to be biologically active in the nanomolar range when used as a synthetic peptide (Supplementary Fig. 7a, Supplementary Table 1, and ref. ^12^). Importantly, deletion of the C-terminal arginine residue in SCOOP12 (SCOOP12Δ1) abolished its activity (Supplementary Fig. 7b), suggesting that the predicted 13-mer corresponds to a minimal bioactive SCOOP epitope. Notably, while MIK2 is the SCOOP12 receptor, a *proscoop12* loss-of-function mutant^12^ did not phenocopy *mik2*, as tested for ROS production and root skewing (Supplementary Fig. 8), suggesting that additional SCOOPs are perceived by MIK2.

**Fig. 3:**
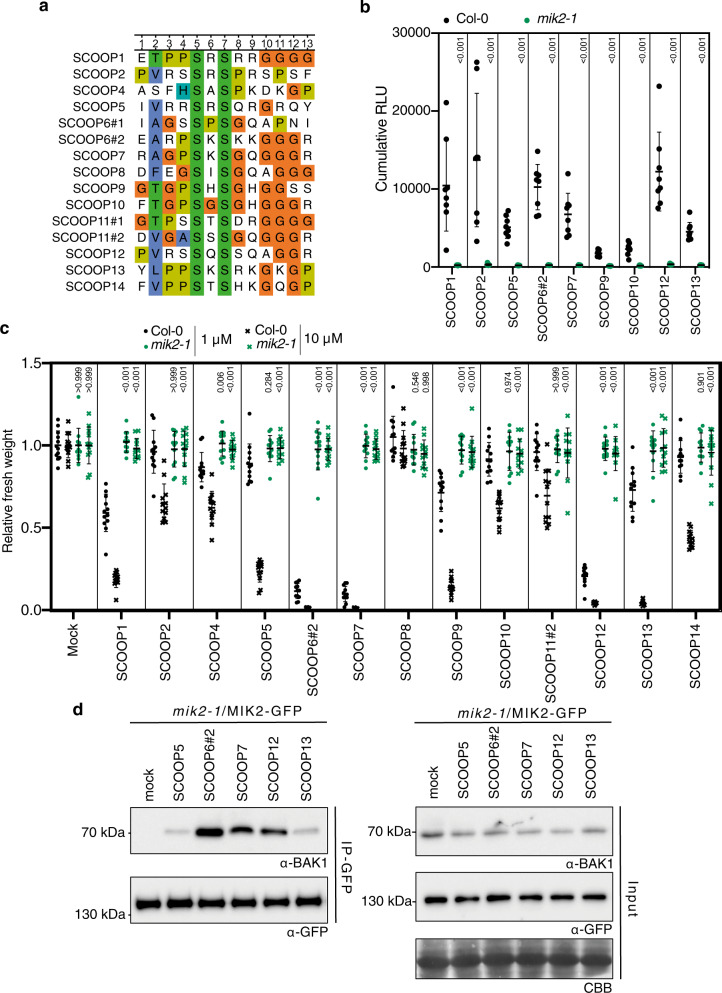
Divergent SCOOP peptides induce MIK2-dependent responses. **a** Alignment of SCOOP motifs from *A. thaliana*. **b** ROS production in leaf disks collected from 4-week-old *A. thaliana* plants induced by application of 1 μM SCOOP peptides in Col-0 (black) or *mik2-1* (green) (*n* = 8 leaf disks). A line represents mean; error bars represent S.D. *P*-values indicate significance relative to the WT control as indicated by two-tailed *T* test **c** Fresh weight of 14-day-old seedlings grown in the presence of 1 μM SCOOP peptides for 10 days relative to mock. A line represents mean; error bars represent S.D. (*n* = 12 seedlings). A two-way ANOVA indicated a significant effect of treatment and genotype (*P* < 0.0001); *P*-values indicate significance relative to Col-0 in a Sidak’s multiple comparison test between genotypes within each treatment. **d** Co-immunoprecipitation of BAK1 with MIK2–GFP from *mik2-1*/MIK2-GFP seedlings treated with 1 μM SCOOP peptides, or water for 10 min. Western blots were probed with antibodies α-GFP and α-BAK1. These experiments were performed and analyzed three times with similar results. These experiments were performed and analyzed three times with similar results. ROS reactive oxygen species.

A multiple alignment of *Arabidopsis* SCOOP sequences corresponding to the defined bioactive epitope revealed poor conservation beyond two conserved serines at positions 5 and 7 (previously shown to be essential for SCOOP12 activity^12^), and the presence of a glycine-rich C-terminal region for SCOOP family members (Fig. 3a). Interestingly, the C-terminal sequence of SCOOP peptides differs from that of other known serine/glycine/proline-rich plant peptides (e.g. CLE, Pep, IDA, PIP, or CEP families^2^), which often contain an arginine–histidine–asparagine motif^17^ suggesting a potential different mode of ligand-binding—something which will need to be tested in the future in structural studies.

We synthesized peptides corresponding to all *Arabidopsis* SCOOPs with available sequence information on TAIR10 (Supplementary Table 1), and found that all but three—SCOOP6, 8, and 11—were active in at least one assay tested at either 1 µM or 10 µM (Fig. 3b, c and Supplementary Fig. 9a, b). Of note, in the absence of current knowledge about the physiological concentration of SCOOP peptides released in the plant apoplast, these arbitrary concentrations were simply chosen to test for the ability of the peptides to induce the responses measured. Notably, upon careful examination of PROSCOOP6 and PROSCOOP11, we noticed additional SCOOP motifs in these proteins^12^—which we named SCOOP6♯2 and SCOOP11♯2 (in contrast to SCOOP6♯1 and SCOOP11♯1 previously tested). Synthetic peptides derived from these alternative SCOOP motifs turned out to be active in multiple assays (Supplementary Fig. 9c–e). Having identified twelve bioactive SCOOP peptides, we then tested if their activities were also MIK2-dependent. Using ROS production and seedling growth inhibition as a readout, we found that, despite the low level of sequence similarity, all active SCOOP peptides required MIK2 (Fig. 3b, c). Furthermore, ligand-induced MIK2–BAK1 association was also observed in response to selected SCOOPs (Fig. 3d). Together, these experiments indicate that MIK2 is the receptor for the divergent family of SCOOP peptides.

The finding that a single receptor is sufficient and necessary for the perception of all members of a given (divergent) peptide family is so far unique to the SCOOP–MIK2 ligand-receptor system. Indeed, members of other plant peptides families (e.g. CLEs, RALFs) are often distinctively or conjunctively perceived by several related receptors^30,31^, or are perceived by partially redundant-related receptors with often distinct quantitative contributions (e.g. perception of CIFs, IDLs, and AtPeps)^2,32,33^.

**Fig. 4:**
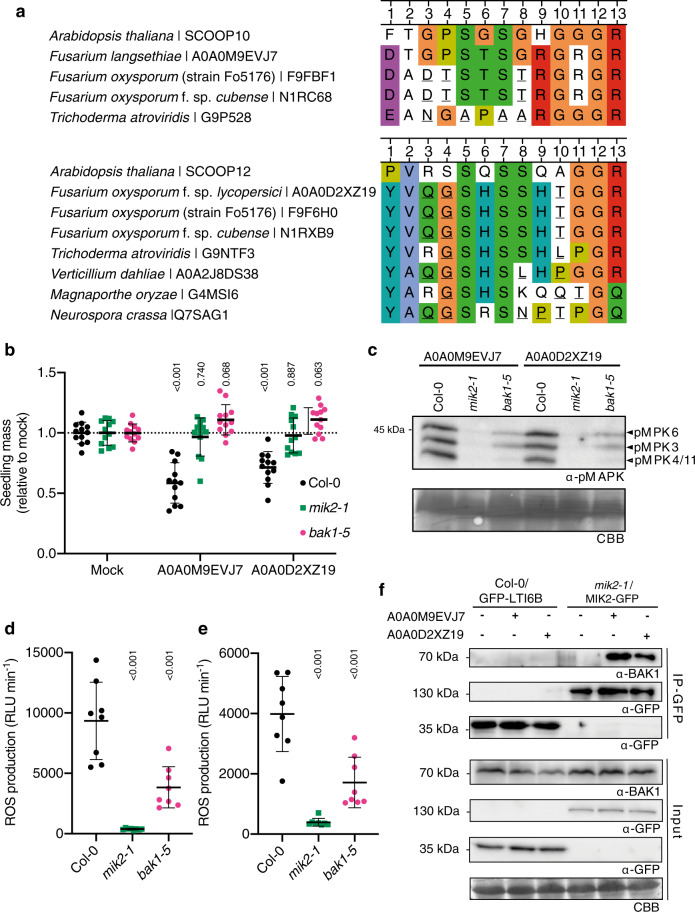
Fusarium-derived peptides are induced MIK2-dependent responses. **a** Alignments of the SCOOP-like motifs from A0A0M9EVJ7 and A0A0D2XZ191 in closely related fungal species with the most closely related *Arabidopsis* SCOOP based on amino acid identity. Residues that are not found in any *Arabidopsis* SCOOP are underlined **b** Relative fresh weight of 14-day-old seedlings grown in the presence of 1 μM A0A0M9EVJ7 or A0A0D2XZ19 for 10 days relative to mock (*n* = 12 seedlings). A line represents mean; error bars represent S.D. *P*-values are derived from a Dunnett’s multiple comparison test comparing the treatment to mock for each genotype following a two-way ANOVA. **c** Western blot using α-p42/p44-ERK recognizing phosphorylated MPK6, MPK3, and MPK4/11 in seedlings treated with 1 μM A0A0M9EVJ7, A0A0D2XZ19 or mock for 15 min. Membranes were stained with CBB, as a loading control. **d**, **e** Integrated ROS production in leaf disks collected from 4-week-old *A. thaliana* plants induced by **d** 1 μM A0A0M9EVJ7 or **e** 1 μM A0A0D2XZ19 application over 40 min (*n* = 8 leaf disks). Line represents mean; error bars represent S.D. *P*-values are derived from a Dunnett’s multiple comparison test comparing the treatment to mock for each genotype following a one-way ANOVA. **f** Co-immunoprecipitation of BAK1 with MIK2–GFP from *mik2-1*/MIK2-GFP and GFP-LTI6B from Col-0/GFP-LTI6B seedlings treated with 1 μM A0A0M9EVJ7, 1 μM A0A0D2XZ19, or water for 10 min. Western blots were probed with antibodies α-GFP and α-BAK1. All experiments were performed and analyzed three times with similar results. CBB Coomassie brilliant blue, ROS reactive oxygen species.

MIK2 is required for resistance to *F. oxysporum*^9^ and is required for immune responses to a *Fusarium* extract^11^. One hypothesis would thus be that perception of the *Fusarium* extract leads to SCOOP perception by MIK2, potentially via the control of PROSCOOP expression, PROSCOOP cleavage, and/or SCOOP secretion. While theoretically possible, this would however not be in line with the responses to the *Fusarium* extract that can be measured within minutes^11^. Homologous sequences to plant peptides have been reported in plant-associated organisms, whose recognition and bioactivity depend on the corresponding plant receptors^34–42^. Using BLAST and MAST algorithms, we identified several SCOOP-like motifs from *Fusarium* proteomes and ordered the corresponding synthetic peptides (Fig. 4a, Supplementary Fig. 10, and Supplementary Table 2). Two of these peptides, derived from the proteins A0A0M9EVJ7 and A0A0D2XZ19, were able to induce immune responses in a MIK2-dependent manner (Fig. 4a–e and Supplementary Fig. 11a–e). Response to these peptides was also BAK1-dependent, and peptide treatment-induced MIK2–BAK1 association (Fig. 4b–f and Supplementary Fig. 11b, c), demonstrating that these synthetic peptides are sufficient as ligands for the MIK2–BAK1 complex. Notably, whilst the SCOOP-like motif present in A0A0M9EVJ7 is relatively poorly conserved, the A0A0D2XZ19 motif shows conservation within related fungal proteomes (Fig. 4a and Supplementary Fig. 11f), consistent with the presence of a MIK2-dependent eliciting activity in extracts from different *Fusarium* species^11^. Together, these results indicate that the elicitor present in *Fusarium* extracts may derive from Fusarium proteins harboring a SCOOP-like motif recognized by MIK2. It however remains to be established in future studies whether these epitope(s) facilitate non-self recognition in the context of an infection, thus contributing to the enhanced susceptibility of *mik2* mutants to *Fusarium*^9,11^.

In conclusion, the characterization of the SCOOP–MIK2 ligand–receptor module represents a significant advance in our understanding of the mechanism through which phytocytokines are perceived by plant cells. Given the multiple physiological functions of MIK2, the identification of SCOOPs as MIK2 ligands will enable the future dissection of the roles of these divergent peptides in diverse pathways and on the molecular basis of their perception.

## Methods

No statistical methods were used to predetermine the sample size. The experiments were not randomized, and investigators were not blinded to allocation during experiments and outcome assessment.

### Plant material and growth conditions

*Arabidopsis thaliana* ecotype Columbia (Col-0) and Wassilewskija (Ws-2) were used as wild-type controls. Plants for ROS burst assays were grown in individual pots at 21 °C with a 10-h photoperiod. Seeds grown on plates were surface sterilized using chlorine gas for 5–6 h, and sown on Murashige and Skoog (MS) media supplemented with vitamins, 1% sucrose, and 0.8% agar and stratified at 4 °C for 2–3 days. *Nicotiana benthamiana* plants were grown on peat-based media at 24 °C, with 16-h photoperiod.

The following *A. thaliana* mutants in Col-0 background (except otherwise indicated) were used: *mik2-1* (SALK_061769)^3^ (kindly provided by Wei-Cai Yang), *mik2-2* (SALK_046987)^9^, *mik2-4* (FLAG_518G04), *bak1-*5^18^, sobir1*−13* (SALK_009453)^21^ (kindly provided by Yuelin Zhang), *cpk28-1* (GK‐523B08)^25^, *rbohD*^23^, *cpk5 cpk6 cpk11*^22^ (kindly provided by Jen Sheen), *bik1 pbl1*^43^ (kindly provided by Jian-Min Zhou), *pro35S::GFP-LTI6B*^44^, and *proscoop12*^12^ (kindly provided by Jean-Pierre Renou). The transgenic lines *efr*/EFR-GFP and *proUBQ10::AEQ* (kindly provided by Justin Lee) lines were described^45,46^. The *mik2-1/pro35S::MIK2–GFP* line was generated in this study. *mik2-1* plants were transformed with the *MIK2* coding sequence in the pEARLEYGATE103 binary vector using floral dip transformation^47^. Transformants were selected to homozygosity using phosphinotricin.

### Synthetic peptides

All synthetic peptides were ordered at >80% purity (physiological assays), >95% purity (biochemical assays) (EZBiolabs). Sequences of all peptides can be found in Supplementary Table 1. The gene models from which the peptide sequences were extracted are listed in Supplementary Table 4.

### Molecular cloning

For overexpression of *MIK2* in *A. thaliana* and *N. benthamiana*, the *MIK2.1* coding sequence was PCR amplified from *Arabidopsis* Col-0 cDNA using gene-specific primers (Supplementary Table 3) and was cloned into pENTR using the D-TOPO kit (Invitrogen) subsequently recombined using LR Clonase II (Invitrogen) into the pEarleygate103 expression vector downstream of the 35S promoter and in frame with a C-terminal poly-His-GFP tag^48^. Generation of the chimeric receptors was performed using overlap extension PCR from EFR and MIK2 entry clones. The final PCR product was then recombined into the pEarleygate103 expression vector using LR Clonase II (Invitrogen). Native promoter sequences were amplified and cloned with C-terminal tags into pICSL86955 using BsaI restriction sites. All clones were verified by Sanger sequencing.

### ROS measurement

Leaf disks were harvested from 4-week-old *Arabidopsis* plants or 3-week-old *N. benthamiana* using a 4-mm diameter biopsy punch (Integra™ Miltex™). Leaf disks were floated overnight on 100 μL of distilled water in white 96-well-plates (Greiner Bio-One). Prior to ROS measurement, the water was removed and replaced with ROS assay solution (100 μM Luminol (Merck), 20 μg mL^−1^ horseradish peroxidase (Merck)) with or without the addition of elicitors. Immediately following the addition of the assay solution light emission was measured from the plate using a HIGH-RESOLUTION PHOTON COUNTING SYSTEM (HRPCS218, Photek) equipped with a 20 mm F1.8 EX DG ASPHERICAL RF WIDE LENS (Sigma Corp).

### Cytoplasmic calcium measurement

*Arabidopsis* seedlings were grown in 96-well plates (Greiner Bio-One) in 100 μL liquid MS for 5 days. The evening before calcium measurements the liquid MS was replaced with 100 μL 20 μM coelenterazine (Merck) and the seedlings incubated in the dark overnight. The following morning the coelenterazine solution was replaced with 100 μL water and rested for a minimum of 30 min in the dark. Readings were taken in a VARIOSKAN^TM^ MUTIPLATE READER (ThermoFisher) using the injector to add 50 μL of 3× concentrated elicitor solution or mock. L_max_ was determined as the maximum luminescence emission upon discharge with (1 M CaCl_2_, 10% ethanol).

### Seedling growth inhibition

Four-day-old seedlings growing on MS plates were transferred individually into separate wells of transparent 48-well tissue culture plates (Greiner Bio-One) containing 500 μL of liquid MS media with/without elicitor addition. The plates were then transferred back to the growth conditions for an additional 10 days before seedlings were blot-dried and weighed.

### Protein extraction and western blot

Two-week-old seedlings grown in liquid MS media (or *N. benthamiana* leaf tissue) were flash-frozen in liquid nitrogen. Plant tissue was ground in liquid nitrogen prior to boiling in 2× Laemmli sample buffer (4% SDS, 20% glycerol, 10% 2-mercaptoethanol, 0.004% bromophenol blue, and 0.125 M Tris-HCl; (10 μL.mg^−1^ tissue)) for 10 min at 95 °C. The samples were then spun at 13,000 × *g* for 5 min prior to loading and running on SDS-PAGE gels of an appropriate concentration. Proteins were transferred onto PVDF membrane (ThermoFisher) prior to incubation with appropriate antibodies (α-pBAK1 (Ser612)^20^ (1:2000); α-GFP-HRP (sc-9996, Santa Cruz; 1:5000); α-pMAPK (p44/42 MAPK (Erk1/2) antibody #9102; 1:4000); α-rabbit-HRP (A-0545, Merck; 1:10,000). Western blots were imaged with a LAS 4000 IMAGEQUANT SYSTEM (GE Healthcare). Staining of the blotted membrane with Coomassie brilliant blue was used to confirm loading.

### Co-immunoprecipitation

Fifteen to twenty seedlings were grown in wells of a 6-well plate for 2 weeks in liquid MS media with gentle agitation (6 wells were used per treatment). The MS media was replaced the night before treatment. Seedlings were treated with 1 μM elf18/SCOOP12 for 10 min before flash-freezing. Tissue was ground and proteins extracted in 1:1 (v/v) powdered tissue:extraction buffer (50 mM Tris-HCl pH 7.5, 150 mM NaCl, 10% glycerol, 5 mM dithiothreitol, 1% protease inhibitor cocktail (P9599, Sigma Aldrich), 2 mM Na_2_MoO_4_, 2.5 mM NaF, 1.5 mM activated Na_3_VO_4_ and 1% IGEPAL) for 30 min at 4 °C. Cell debris was removed via centrifugation. For immunoprecipitation, GFP-TRAP/RFP-TRAP AGAROSE BEADS (ChromoTek) were incubated with extracts for 3 h at 4 °C and washed three times in wash buffer (50 mM Tris-HCl pH 7.5, 150 mM NaCl, 10% glycerol, 5 mM dithiothreitol, 1% protease inhibitor cocktail (P9599, Sigma Aldrich), 2 mM Na2MoO4, 2.5 mM NaF, 1.5 mM activated Na_3_VO_4_, and 0.1% IGEPAL) before adding 2× Laemmli sample buffer and incubating for 10 min at 95 °C. Detection was carried out by SDS-PAGE and western blots using α-BAK1^19^ α-RFP (ab34771, Abcam) and α-GFP-HRP (sc-9996, Santa Cruz) antibodies. α-rabbit-HRP (A-0545, Merck) was used to detect α-BAK1 and α-RFP.

### Transient expression in *Nicotiana benthamiana*

*Agrobacterium tumefaciens* strain GV3101 transformed with the corresponding construct were grown overnight in L-media and spun-down. For ROS assays the bacteria were resuspended in 10 mM MgCl_2_ and adjusted to O.D._600_ = 0.2 prior to infiltration into the youngest fully expanded leaves of 3-week-old plants. Leaf disks were collected 24 h later, and ROS assays were performed as described for *Arabidopsis*.

### Root skewing

Seeds were sown directly on MS agar square plates and stratified for 2 days at 4 °C. Plates were transferred to 22 °C under a 16-h photoperiod, in an upright position for 9 days. The root angle was measured by the ImageJ software, as performed previously^9^.

### Protein expression and purification

Codon-optimized synthetic genes for expression in *Spodoptera frugiperda* (Invitrogen GeneArt), coding for *Arabidopsis thaliana* MIK2 (residues 1–709) and BAK1 (1–220) ectodomains were cloned into a modified pFastBac (Geneva Biotech) vector, providing a TEV (tobacco etch virus protease) cleavable C-terminal StrepII-9xHis tag. For protein expression, *Trichoplusia ni* Tnao38 cells^49^ were infected with MIK2 or BAK1 virus with a multiplicity of infection (MOI) of 3 and incubated for 1 d at 28 °C and 2 d at 22 °C at 110 rpm. The secreted proteins were purified from the supernatant by sequential Ni^2+^ (HisTrap Excel column; GE Healthcare; equilibrated in 25 mM KP_i_ pH 7.8, 500 mM NaCl) and StrepII (Strep-Tactin Superflow high capacity (IBA Lifesciences) equilibrated in 25 mM Tris pH 8.0, 250 mM NaCl, 1 mM EDTA) affinity chromatography. All proteins were incubated with TEV protease to remove the tags. Proteins were further purified by SEC on a Superdex 200 increase 10/300 GL column (GE Healthcare), equilibrated in 20 mM sodium citrate pH 5.0, 150 mM NaCl. For biochemical experiments, proteins were concentrated using Amicon Ultra concentrators (Millipore, molecular weight cutoff 10,000 and 30,000).

### ITC experiments

Experiments were performed at 25 °C using a MicroCal PEAQ-ITC (Malvern Instruments) with a 200-µL standard cell and a 40-μL titration syringe. MIK2 and BAK1 ectodomains were gel filtrated into pH 5 ITC buffer (20 mM sodium citrate pH 5.0, 150 mM NaCl). SCOOP12 peptide powder was dissolved in the same buffer to obtain the desired concentration. A typical experiment consisted of injecting 1 μL of a 200 μM solution of the ligand into 20 μM MIK2 solution in the cell at 150 s intervals. BAK1 vs MIK2–SCOOP12 experiments were performed by titrating 200 µM BAK1 onto the MIK2–SCOOP12 complex in the cell, using the same injection pattern. ITC data were corrected for the heat of dilution by subtracting the mixing enthalpies for titrant solution injections into protein-free ITC buffer. Experiments were done in triplicates and data were analyzed using the MicroCal PEAQ-ITC Analysis Software provided by the manufacturer. All ITC runs used for data analysis had an *N* ranging between 0.8 and 1.3. The *N* values were fitted to 1 in the analysis.

### Similarity/identity matrices

SIAS was used to generate similarity and identity matrices (http://imed.med.ucm.es/Tools/sias.html).

### Species divergence estimations

The timing of fungal species divergence was estimated using TimeTree^50–53^.

### Statistical analysis

Statistical analysis was performed in GraphPad Prism 7.0. (GraphPad Software, http://www.graphpad.com) unless stated otherwise. Dot plots were used to show individual data points wherever possible. *P-*values < 0.05 were considered nonsignificant. Sample sizes, statistical tests used, and *P-*values are stated in the figure legends.

### Reporting summary

Further information on research design is available in the Nature Research Reporting Summary linked to this article.

## Supplementary information

Supplementary Information

Peer Review File

Reporting Summary

## Data Availability

For blot source images, see Supplementary Fig. 1. All other data or materials can be obtained from the corresponding author upon request. Source data are provided with this paper.

## Source data

Source Data
