# Supplementary material for: Perception of a divergent family of phytocytokines by the Arabidopsis receptor kinase MIK2
Source: Nat Commun. 2021 Jan 29;12:705. doi: 10.1038/s41467-021-20932-y (PMC7846792; doi:10.1038/s41467-021-20932-y)
Supplement: Supplementary file 1 — Supplementary Information [file 41467_2021_20932_MOESM1_ESM.pdf]

Perception of a divergent family of phytocytokines by the Arabidopsis receptor kinase MIK2

Rhodes *et al.*

Supplementary Figures 1 – 11; Supplementary Tables 1 - 4

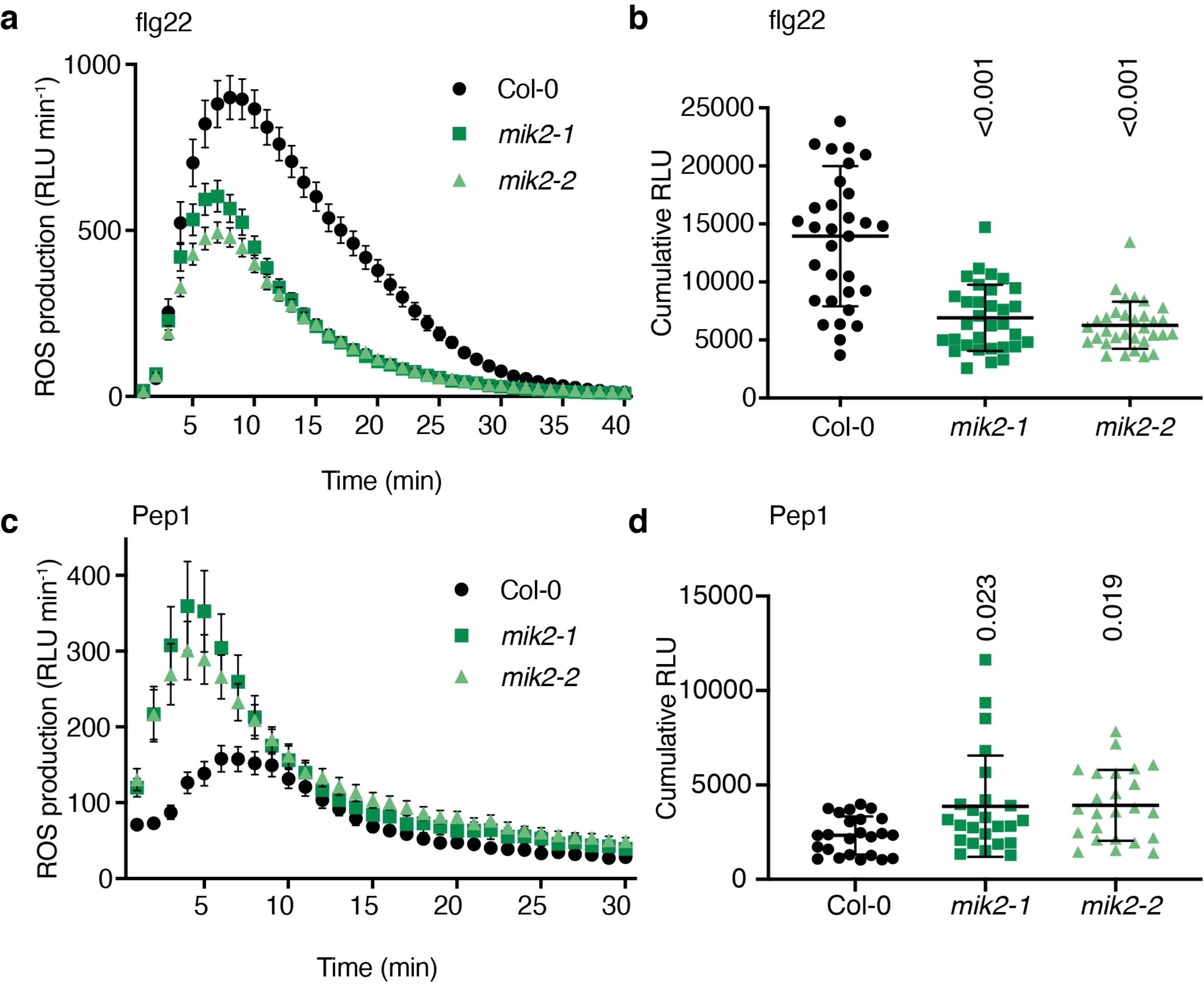

Supplementary Figure 1. Differential regulation of ROS production in response to flg22 and Pep1 in *mik2* plants.
(**a** to **d**) ROS production in leaf discs collected from 4-week-old *A. thaliana* plants induced by 100 nM flg22 (**a** and **b**) (*n*=32 leaf disks) or 1 µM Pep1 (**c** and **d**) (*n*=24 leaf disks). (**a** and **c**) Points represent mean; error bars represent S.E.M. (**b** and **d**) Integrated ROS production over 40 min (**b**) and 30 min (**d**) from (**a**) and (**c**) respectively. Line represents mean; error bars represent S.D. P-values indicate significance in a Tukey's multiple comparisons test following one-way ANOVA. Experiments have been repeated and analyzed three times with similar results.

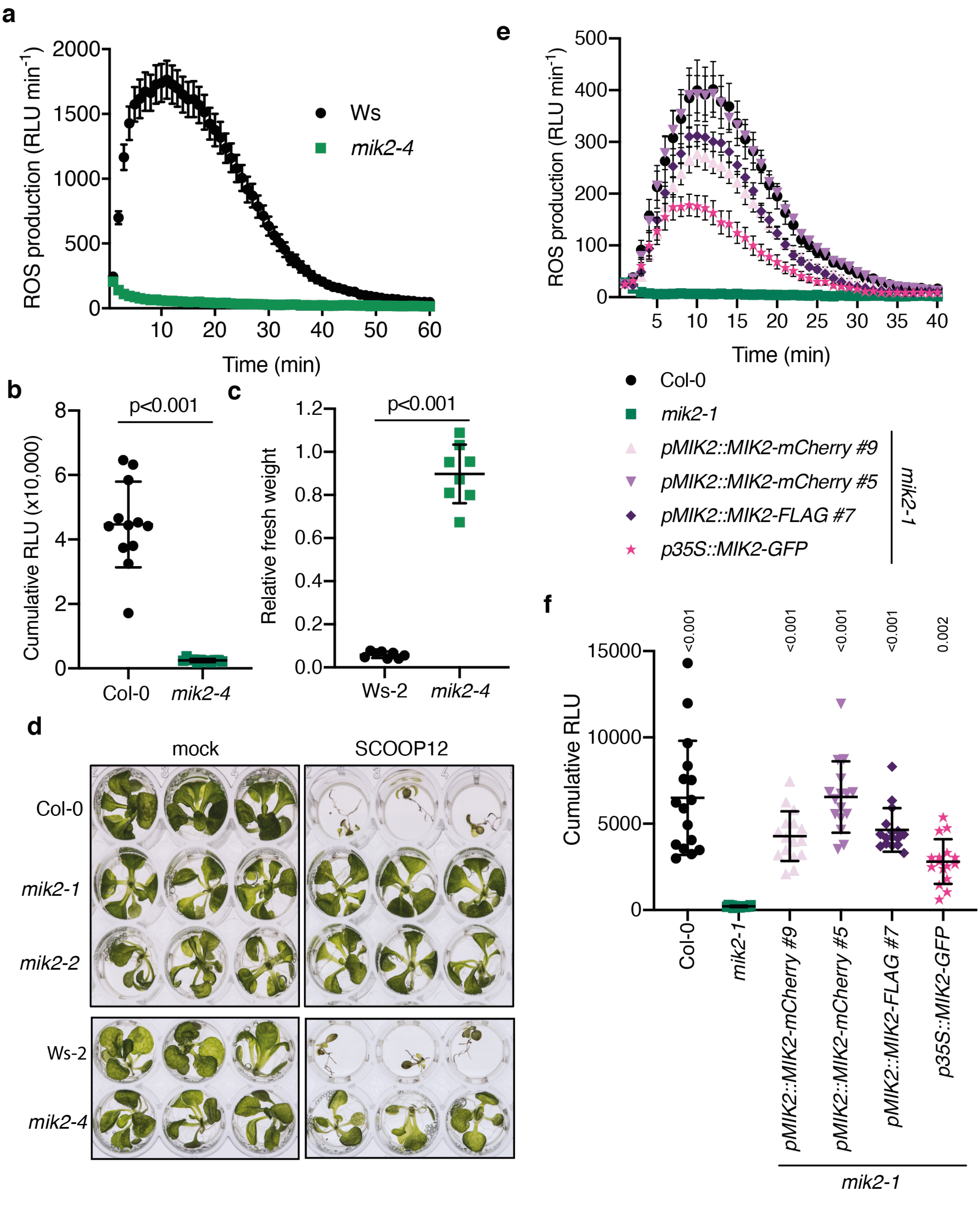

Supplementary Figure 2. SCOOP12-induced responses are MIK2-dependent.
(**a** and **b**) ROS production in leaf discs collected from 4-week-old *A. thaliana* plants induced by 1 µM SCOOP12 (*n*=12 leaf disks). (**c**) Fresh weight and (**d**) Images of 14-day-old seedlings grown in the presence of 1 µM SCOOP12 for 10 days relative to mock (*n*=8 seedlings). Line represents mean; error bars represent S.D. P-values indicate the results of two-tailed T-tests. Experiments were repeated and analyzed twice (Ws ecotype) or three times (Col-0 ecotype) with similar results. (**e** to **f**) ROS production in leaf discs collected from 4-week-old *A. thaliana* plants induced by 100 nM SCOOP12 (*n*=16 leaf disks). (**a** and **e**) Points represent mean; error bars represent S.E.M. (**b** and **f**) Integrated ROS production over 40 min from (**a** and **e**). Line represents mean; error bars represent S.D. P-values indicate significance relative to *mik2-1* in a Tukey's multiple comparisons test following one-way ANOVA.

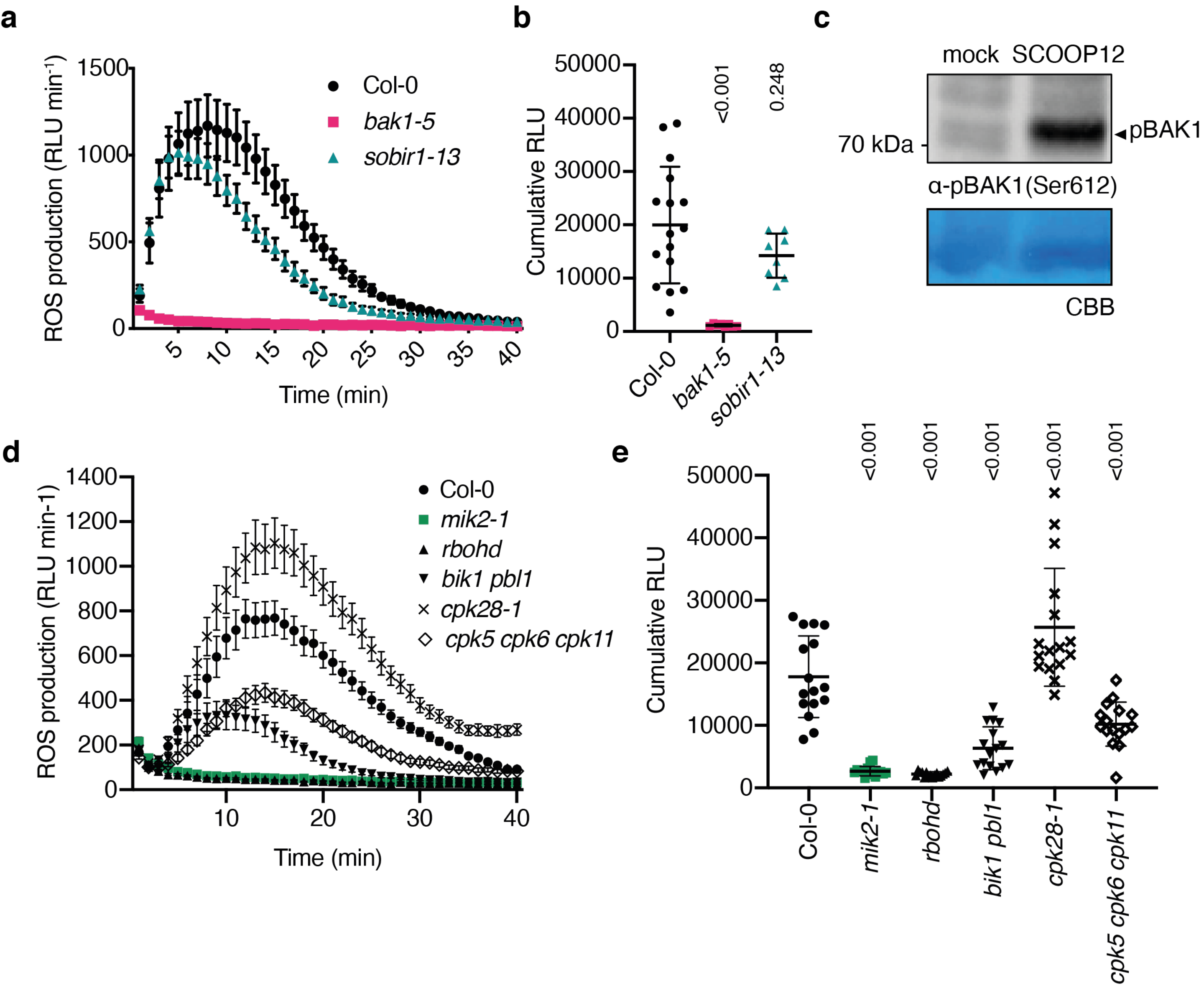

Supplementary Figure 3. SCOOP12-induced responses are SERK-dependent and SOBIR1-independent. SCOOP12 induces ROS production through a pathway conserved with other LRR-RK pattern-recognition receptors.
(**a** and **b**) ROS production in leaf discs collected from 4-week-old *A. thaliana* plants induced by 1 µM SCOOP12 (*n*=16 leaf disks). (**c**) Western blot using α-pBAK1(Ser612) of seedlings after 15 min treatment with 100 nM SCOOP. (**d** and **e**) ROS production in leaf discs collected from 4-week-old *A. thaliana* plants induced by 1 µM SCOOP12 (*n*=16 leaf disks). (**a** and **d**) Points represent mean; error bars represent S.E.M. (**b** and **e**) Integrated ROS production over 40 min from (**a** and **d**). Line represents mean; error bars represent S.D. P-values indicate significance relative to Col-0 in a Tukey's multiple comparisons test following one-way ANOVA. Experiments were repeated and analyzed three times with similar results. See Material and Methods for genotype references.

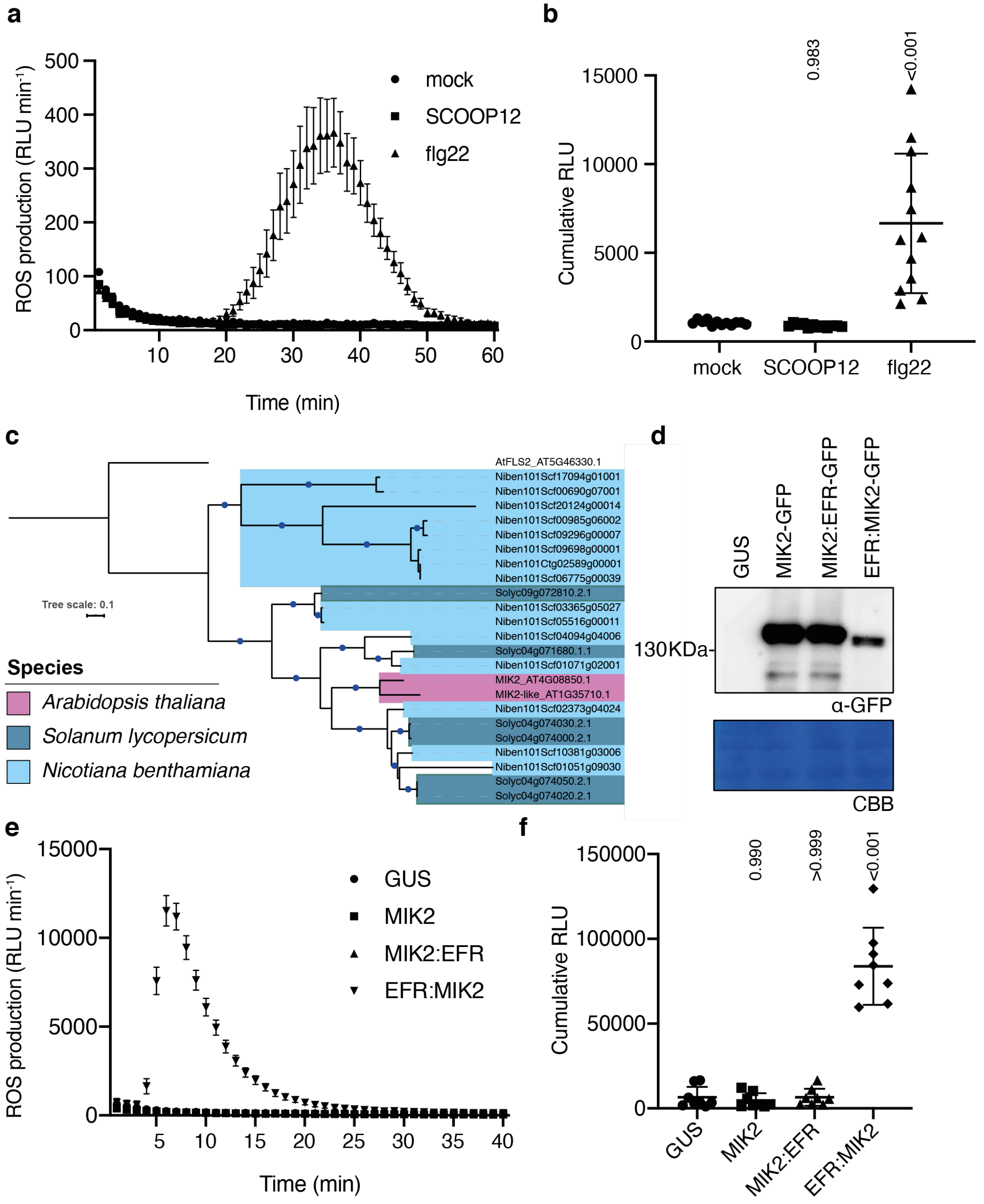

Supplementary Figure 4. SCOOP12 is unable to induce ROS production in *N. benthamiana*, which lacks a *MIK2* ortholog.

(**a** and **b**) ROS production in leaf discs collected from 3-week-old *N. benthamiana* plants induced by 1 µM SCOOP12, 1 µM flg22 or mock (*n*=12 leaf disks). (**a**) Points represent mean; error bars represent S.E.M. (**b**) Integrated ROS production over 40 min from (**a**). Line represents mean; error bars represent S.D. (**c**) Phylogenetic tree based on LRR-RK subfamily XIIb proteins (as defined in refs. *41* and *42*). Amino acid sequences of the kinase domains were aligned using MUSCLE and a tree was generated using PhyML maximum likelihood with LG matrix. The tree was visualized using iTOL. Blue dots indicate nodes with >80% bootstrapping support (100 iterations performed). (**d**) Western blot using α-GFP to confirm the accumulation of the respective proteins. CBB is shown as a loading control. (**e** and **f**) ROS production induced by 100 nM elf18 in *N. benthamiana* leaf disks transiently expressing the defined constructs (*n*=8 leaf disks). (**e**) Points represent mean; error bars represent S.E.M. (**f**) Integrated ROS production over 40 min from (**e**). Line represents mean; error bars represent S.D. P-values indicate significance relative to mock or GUS in a Tukey's multiple comparisons test following one-way ANOVA. Experiments were repeated and analyzed three times with similar results.

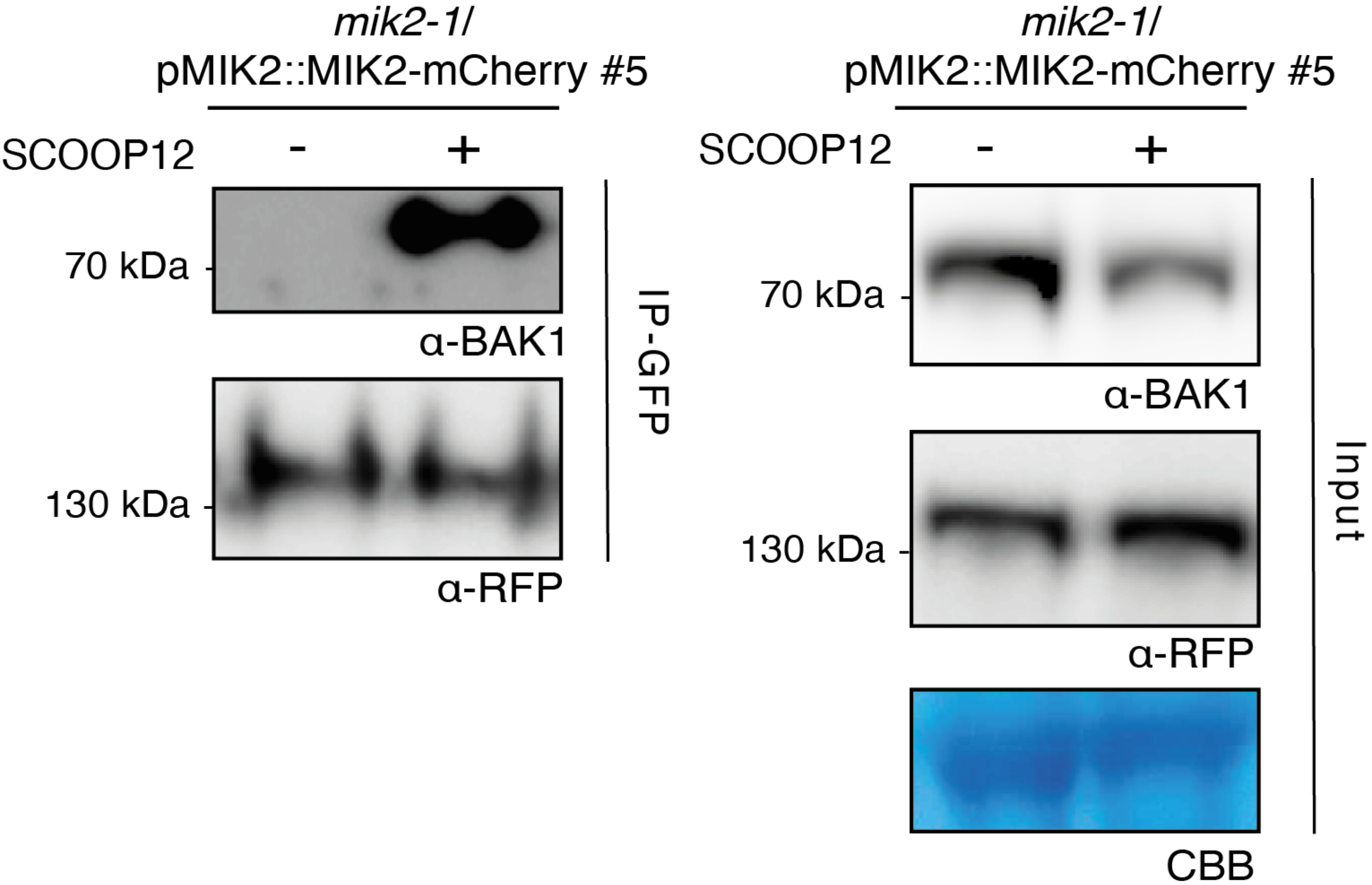

Supplementary Figure 5. SCOOP12-induced MIK2-BAK1 complex formation is observable when MIK2 is expressed under its native promoter

(**a**) Co-immunoprecipitation of BAK1 with MIK2-mCherry from *mik2-1*/pMIK2::MIK2-mCherry seedlings treated with 1 μM SCOOP12, or water for 10 min. Western blots were probed with antibodies α-RFP and α-BAK1. Experiments were repeated three times with similar results.

a

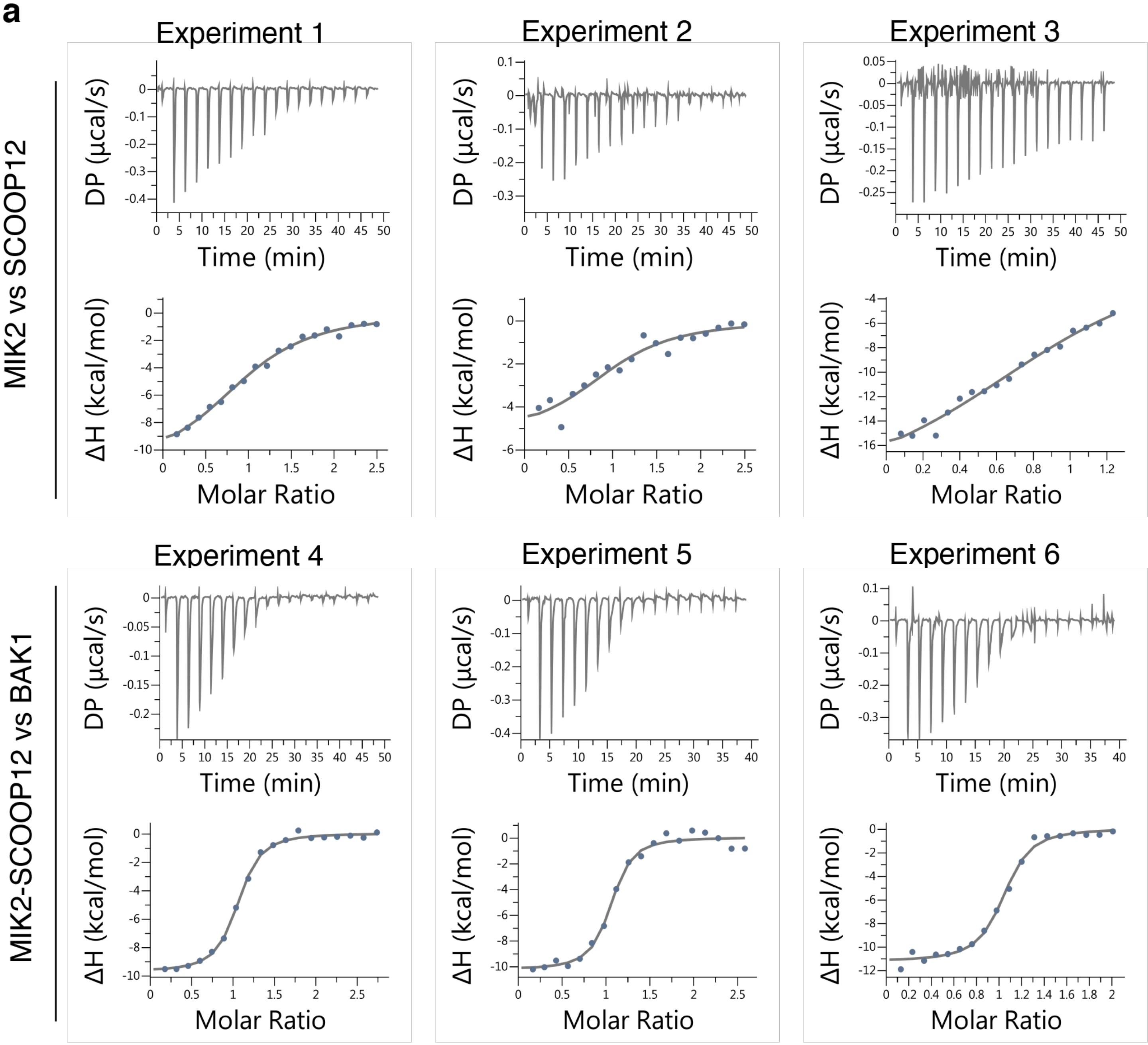

b

Experiment	Ligand (Syringe)	Protein (Cell)	pH	Kd (nM)	ΔH (kcal/mol)	N
1	SCOOP12	MIK2	5	5110 ± 1410	-11.5 ± 1.4	1
2	SCOOP12	MIK2	5	4080 ± 3920	-5.40 ± 2.2	1
3	SCOOP12	MIK2	5	4490 ± 3970	-20.2 ± 9.1	1
4	BAK1	MIK2-SCOOP12	5	267 ± 37	-9.8 ± 0.2	1
5	BAK1	MIK2-SCOOP12	5	273 ± 66	-11.3 ± 0.4	1
6	BAK1	MIK2-SCOOP12	5	286 ± 92	-10.3 ± 0.4	1

Supplementary Figure 6. Independent ITC experiments of MIK2 vs SCOOP12, and MIK2-SCOOP12 vs BAK1.

(**a**) ITC raw thermograms. Experiments 1, 2 and 3 are ITC runs of MIK2 vs SCOOP12. Experiments 4, 5 and 6 are ITC runs of MIK2-SCOOP12 vs BAK1. (**B**) Table summaries reporting the values measured in each independent experiment. K_d indicates the binding affinity in nanomolar, DH is the enthalpy and N indicates the number of sites (N = 1 for a 1:1 interaction).

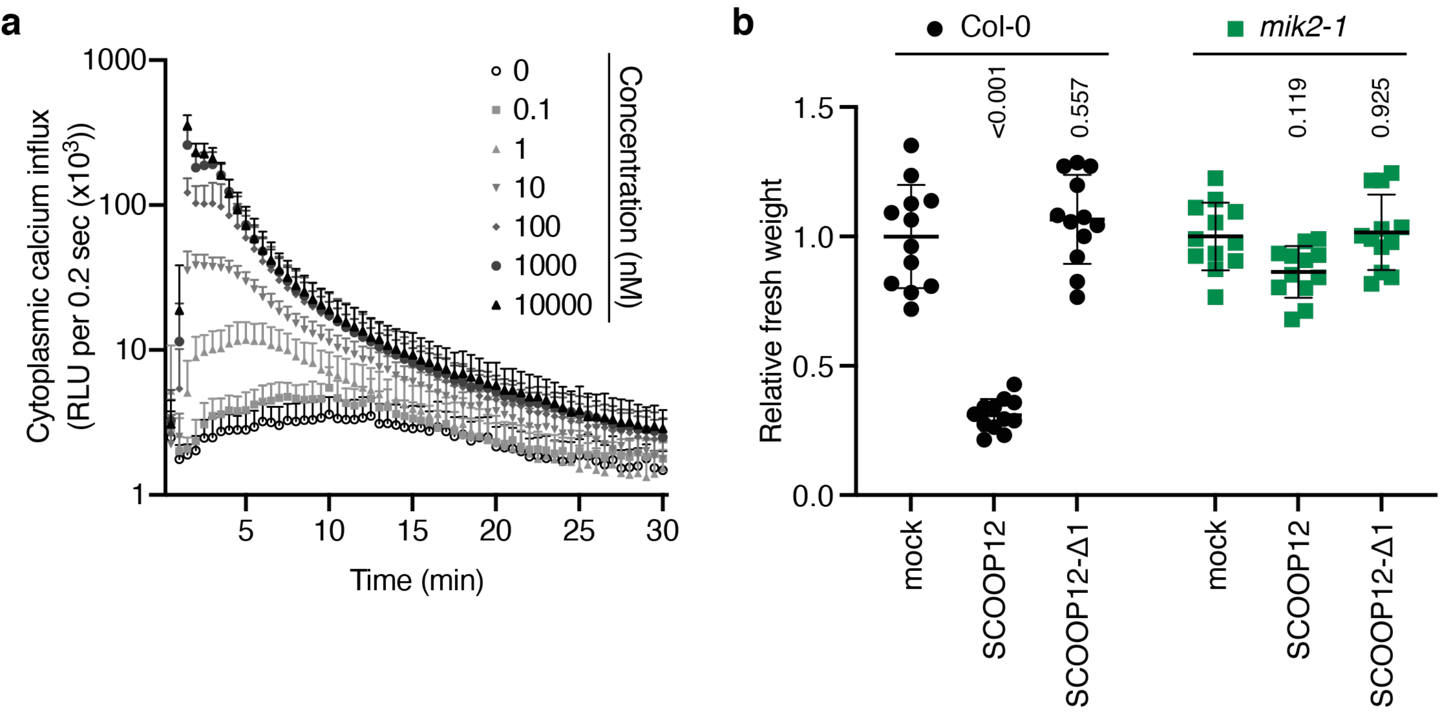

Supplementary Figure 7. SCOOP12 has discernable activity in the nanomolar range. The C-terminal residue of SCOOP12 is essential for activity.

(**a**) SCOOP12-induced cytoplasmic calcium influx measured as RLU in Col-0$^{35S::AEQ}$ seedlings (n=12 seedlings). Points represent mean; error bars represent S.E.M. (**b)** Fresh weight of 14-day-old seedlings grown in the presence of 1 μM SCOOP12 or SCOOP12 Δ1 for 10 days relative to mock. Line represents mean; error bars represent S.D. P-values indicate significance relative to mock in a Tukey's multiple comparisons test following one-way ANOVA. Experiments were repeated and analyzed three times with similar results.

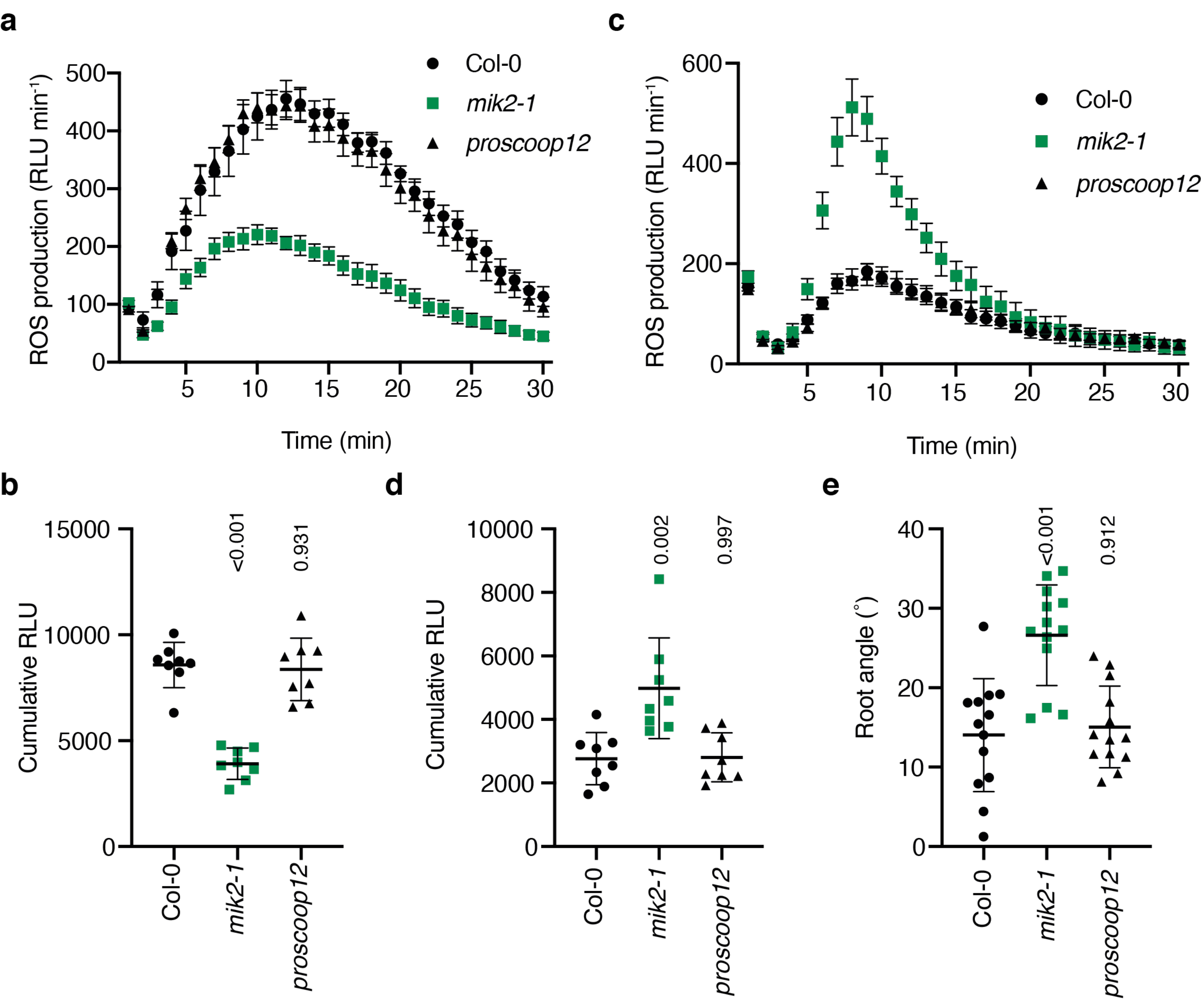

Supplementary Figure 8. The *proscoop12* mutant does not phenocopy *mik2*.
(**a** to **d**) ROS production in leaf discs collected from 4-week-old *A. thaliana* plants induced by 100 nM flg22 (**a** and **b**) and 1 µM Pep1 (**c** and **d**) (*n*=8 leaf disks). (**a** and **c**) Points represent mean; error bars represent S.E.M. (**b** and **d**) Integrated ROS production over 30 min. Line represents mean; error bars represent S.D. (**e**) Quantification of the root angle of 9-day-old seedlings grown in an upright position on MS agar medium (*n*=13 seedlings). Lines represents mean; error bars represent S.D. P-values indicate significance relative to Col-0 in a Tukey's multiple comparisons test following one-way ANOVA. Experiments were repeated and analyzed three times with similar results.

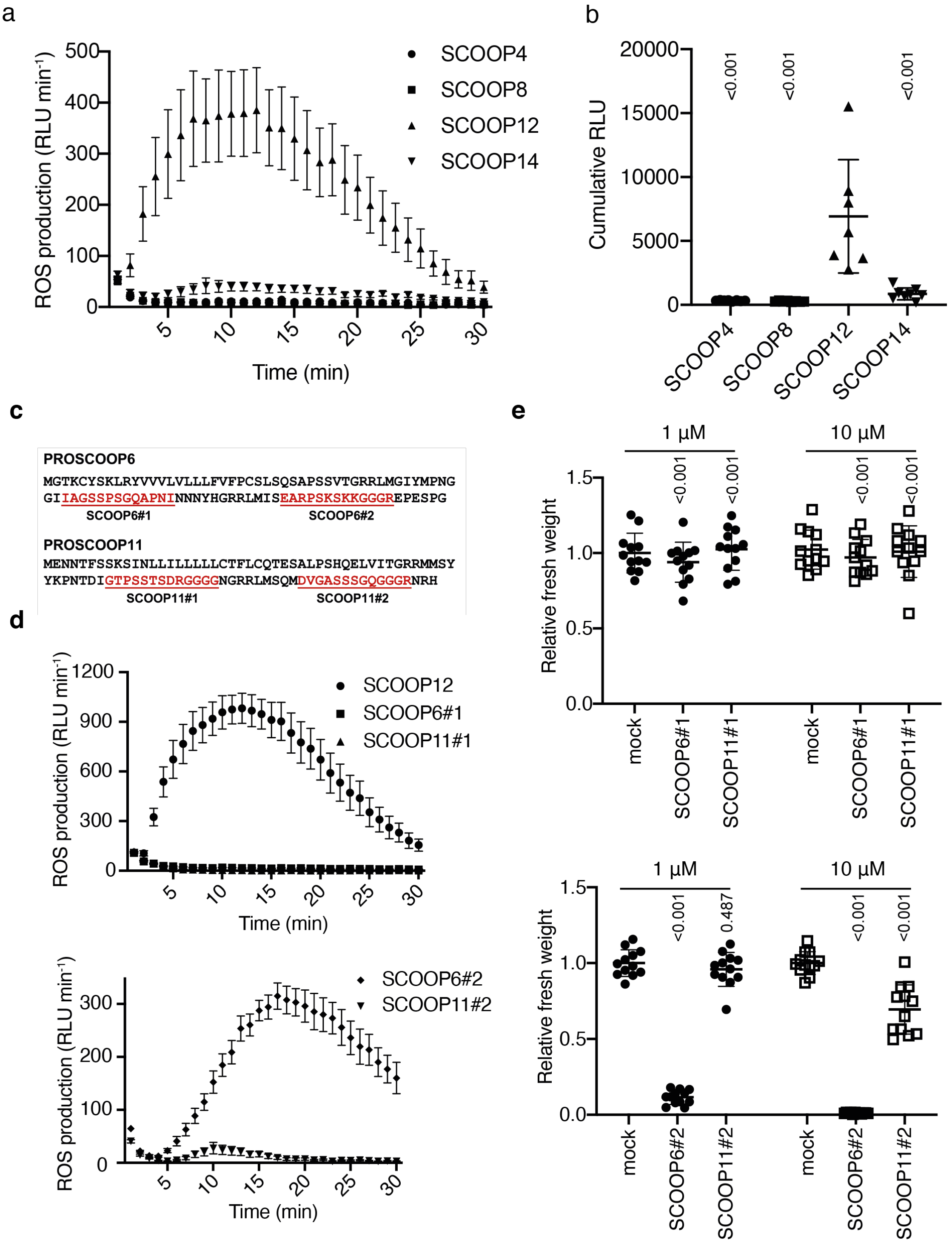

Supplementary Figure 9. SCOOP4, SCOOP8 and SCOOP14 do not elicit detectable ROS production in Col-0. PROSCOOP6 and PROSCOOP11 contain two SCOOP motifs with only one being active.

ROS production in leaf discs collected from 4-week-old *A. thaliana* plants induced by 1 µM SCOOP4, SCOOP8, SCOOP12 and SCOOP14 (*n*=8 leaf disks). (**b**) Integrated ROS production over 40 min. Lines represents mean; error bars represent S.D. Experiments were repeated and analyzed three times with similar results. (**c**) Amino acid sequence of PROSCOOP6 and PROSCOOP11 with two SCOOP motifs. (**d**) ROS production induced by 1 µM SCOOP in leaf disks from 4-week-old plants (*n*=8 leaf disks). (**a** and **d**) Points represent mean; error bars represent S.E.M. (**e**) Fresh weight of 14-day-old seedlings grown in the presence of 1 µM or 10 µM SCOOP for 10 days relative to mock (*n*=12 seedlings). Lines represent mean; error bars represent S.D. P-values indicate significance relative to SCOOP12 or mock treatment in a Tukey's multiple comparisons test following one-way ANOVA, $p<0.01$. Experiments were repeated and analyzed three times with similar results.

a

	SCOOP1	SCOOP2	SCOOP4	SCOOP5	SCOOP6#2	SCOOP7	SCOOP8	SCOOP9	SCOOP10	SCOOP11#2	SCOOP12	SCOOP13	SCOOP14
SCOOP1	100.0%												
SCOOP2	30.8%	100.0%											
SCOOP4	23.1%	23.1%	100.0%										
SCOOP5	38.5%	46.2%	15.4%	100.0%									
SCOOP6#2	53.8%	23.1%	30.8%	30.8%	100.0%								
SCOOP7	46.2%	15.4%	23.1%	23.1%	69.2%	100.0%							
SCOOP8	38.5%	15.4%	23.1%	15.4%	30.8%	46.2%	100.0%						
SCOOP9	46.2%	23.1%	15.4%	23.1%	38.5%	53.8%	30.8%	100.0%					
SCOOP10	53.8%	15.4%	23.1%	23.1%	53.8%	69.2%	38.5%	69.2%	100.0%				
SCOOP11#2	38.5%	23.1%	23.1%	30.8%	46.2%	69.2%	53.8%	46.2%	61.5%	100.0%			
SCOOP12	30.8%	46.2%	23.1%	30.8%	46.2%	46.2%	46.2%	23.1%	38.5%	53.8%	100.0%		
SCOOP13	53.8%	15.4%	46.2%	23.1%	53.8%	46.2%	23.1%	30.8%	38.5%	30.8%	23.1%	100.0%	
SCOOP14	46.2%	23.1%	38.5%	30.8%	46.2%	38.5%	23.1%	30.8%	46.2%	38.5%	30.8%	61.5%	100.0%
RKL12880.1	23.1%	46.2%	15.4%	23.1%	23.1%	30.8%	23.1%	15.4%	23.1%	38.5%	69.2%	23.1%	30.8%
RKK91479.1	23.1%	46.2%	15.4%	23.1%	23.1%	23.1%	23.1%	23.1%	23.1%	23.1%	61.5%	15.4%	15.4%
KIL85672.1	23.1%	23.1%	23.1%	15.4%	23.1%	38.5%	38.5%	23.1%	23.1%	30.8%	61.5%	23.1%	23.1%
A0A0D2XZ19	30.8%	23.1%	23.1%	23.1%	38.5%	38.5%	38.5%	38.5%	46.2%	46.2%	53.8%	30.8%	30.8%
I1RWN2	53.8%	46.2%	23.1%	38.5%	30.8%	38.5%	30.8%	30.8%	38.5%	38.5%	30.8%	46.2%	38.5%
X0K0D8	53.8%	30.8%	23.1%	38.5%	38.5%	38.5%	30.8%	30.8%	38.5%	38.5%	30.8%	30.8%	30.8%
A0A0D2XQW0	46.2%	38.5%	23.1%	23.1%	38.5%	38.5%	30.8%	53.8%	46.2%	38.5%	38.5%	38.5%	46.2%
A0A0D2XVW5	46.2%	23.1%	15.4%	30.8%	38.5%	46.2%	30.8%	46.2%	46.2%	38.5%	23.1%	30.8%	30.8%
A0A098DTX8	38.5%	15.4%	15.4%	23.1%	30.8%	46.2%	38.5%	53.8%	53.8%	38.5%	30.8%	23.1%	23.1%
A0A0M9EVJ7	53.8%	23.1%	23.1%	38.5%	46.2%	61.5%	38.5%	53.8%	69.2%	61.5%	30.8%	38.5%	46.2%
RKK80373.1	46.2%	23.1%	30.8%	23.1%	46.2%	61.5%	38.5%	46.2%	53.8%	46.2%	30.8%	38.5%	38.5%
F9FC73	46.2%	38.5%	23.1%	38.5%	46.2%	30.8%	30.8%	23.1%	30.8%	30.8%	46.2%	30.8%	30.8%
F9G0P6	46.2%	38.5%	23.1%	23.1%	30.8%	38.5%	30.8%	38.5%	38.5%	30.8%	30.8%	38.5%	30.8%

b

	SCOOP1	SCOOP2	SCOOP4	SCOOP5	SCOOP6#2	SCOOP7	SCOOP8	SCOOP9	SCOOP10	SCOOP11#2	SCOOP12	SCOOP13	SCOOP14
SCOOP1	1	0.1	0.02	0.17	0.59	0.43	0.27	0.35	0.36	0.31	0.23	0.5	0.37
SCOOP2	0.09	1	0.05	0.35	0.13	-0.01	-0.02	0.01	-0.1	0.04	0.39	0	-0.01
SCOOP4	0.02	0.06	1	-0.03	0.1	0.02	-0.04	-0.06	0	0	0	0.23	0.18
SCOOP5	0.15	0.34	-0.02	1	0.19	0.02	-0.13	0	0	0.06	0.14	0.16	0.14
SCOOP6#2	0.56	0.13	0.1	0.2	1	0.63	0.17	0.24	0.37	0.43	0.47	0.41	0.28
SCOOP7	0.42	-0.01	0.02	0.03	0.65	1	0.26	0.47	0.6	0.68	0.41	0.3	0.21
SCOOP8	0.26	-0.03	-0.04	-0.14	0.17	0.26	1	0.13	0.14	0.45	0.34	0	0.01
SCOOP9	0.36	0.01	-0.07	0	0.26	0.5	0.14	1	0.61	0.42	0.12	0.11	0.1
SCOOP10	0.38	-0.12	0	0	0.41	0.65	0.16	0.62	1	0.59	0.25	0.25	0.29
SCOOP11#2	0.29	0.04	0	0.06	0.43	0.65	0.44	0.37	0.52	1	0.5	0.13	0.18
SCOOP12	0.21	0.37	0	0.14	0.44	0.37	0.32	0.1	0.21	0.48	1	0.08	0.08
SCOOP13	0.5	0	0.24	0.18	0.44	0.31	0	0.1	0.24	0.15	0.09	1	0.67
SCOOP14	0.39	-0.01	0.2	0.17	0.31	0.23	0.01	0.1	0.29	0.21	0.09	0.69	1
RKL12880.1	0.08	0.28	-0.1	0.04	0.07	0.11	0	-0.04	-0.02	0.21	0.55	0.09	0.09
RKK91479.1	0.11	0.39	0	0.12	0.14	0.08	0.08	0.13	0.01	0.12	0.57	-0.01	-0.04
KIL85672.1	0.08	0.13	-0.01	0.04	0.14	0.21	0.19	0.06	-0.01	0.19	0.52	0.11	0.08
A0A0D2XZ19	0.14	0.07	-0.05	0.03	0.26	0.23	0.27	0.27	0.37	0.31	0.49	0.13	0.1
I1RWN2	0.42	0.37	0.01	0.18	0.25	0.33	0.17	0.14	0.18	0.34	0.23	0.38	0.27
X0K0D8	0.35	0.09	0.12	0.25	0.31	0.28	0.05	0.14	0.22	0.21	0.23	0.16	0.13
A0A0D2XQW0	0.4	0.24	0.02	0.04	0.25	0.28	0.23	0.37	0.26	0.37	0.34	0.2	0.28
A0A0D2XVW5	0.28	0	-0.08	0.15	0.26	0.36	0.05	0.32	0.29	0.19	0.07	0.15	0.1
A0A098DTX8	0.25	-0.01	-0.07	0.04	0.2	0.42	0.22	0.47	0.41	0.34	0.23	0.02	0
A0A0M9EVJ7	0.42	0.01	0.1	0.15	0.43	0.57	0.23	0.43	0.56	0.63	0.2	0.27	0.35
RKK80373.1	0.28	-0.03	0.14	-0.01	0.31	0.52	0.13	0.33	0.4	0.31	0.17	0.25	0.21
F9FC73	0.32	0.22	0.08	0.21	0.4	0.2	0.3	0.08	0.12	0.31	0.44	0.12	0.14
F9G0P6	0.42	0.22	0.08	0.01	0.22	0.33	0.2	0.21	0.2	0.25	0.25	0.27	0.2

Supplementary Figure 10. Similarity matrices of *Arabidopsis* SCOOP peptides and SCOOP-like peptides from *Fusarium* proteomes

(**a** and **b**) Similarity matrices showing (**a**) percentage identity and (**b**) a normalized similarity score generated using the BLOSUM62 matrix. Active *Fusarium* epitopes are outlined.

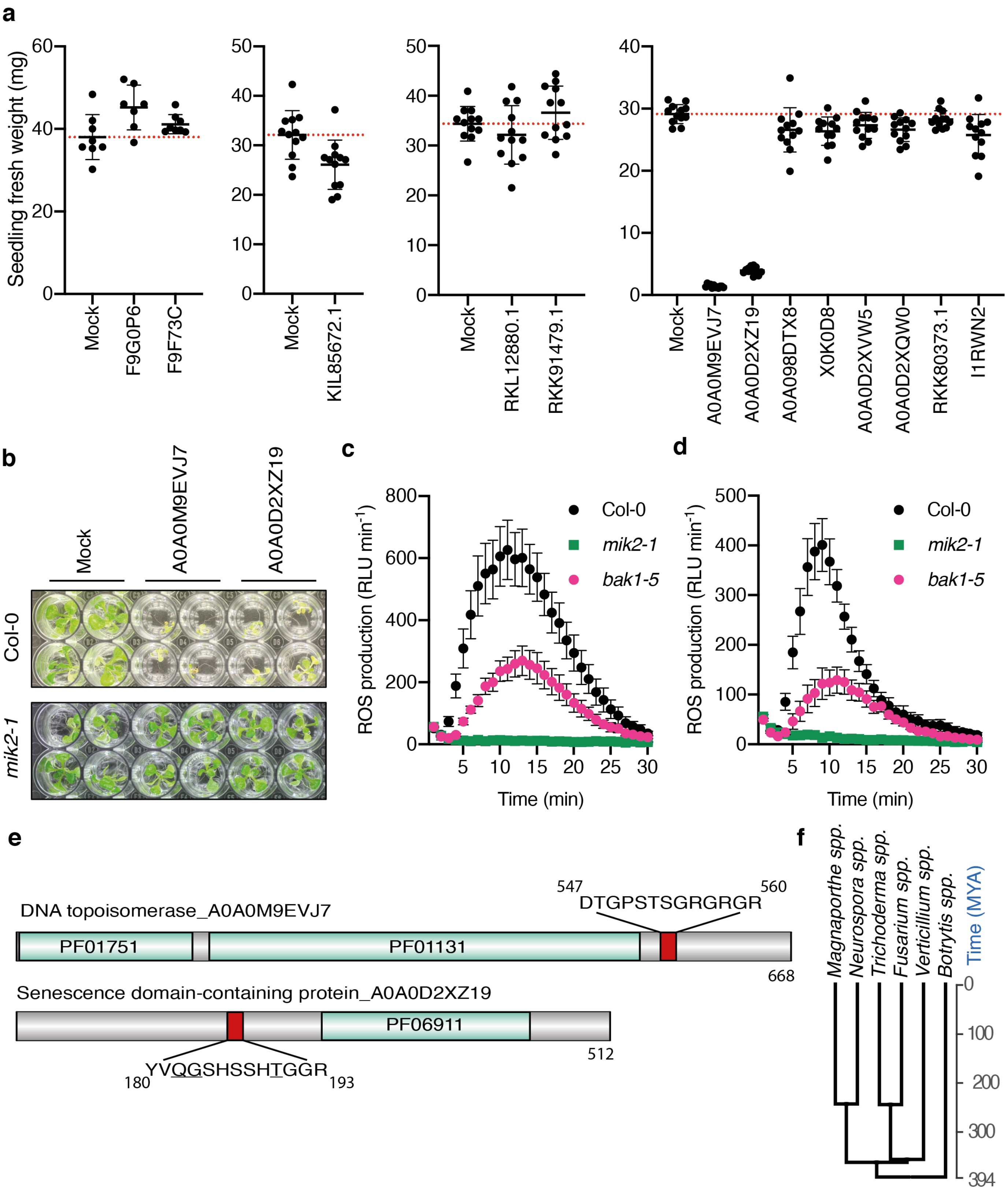

a
Seedling fresh weight (mg)
Mock
F9G0P6
F9F73C
Mock
KIL85672.1
Mock
RKL12880.1
RKK91479.1
Mock
A0A0M9EVJ7
A0A0D2XZ19
A0A098DTX8
X0K0D8
A0A0D2XVW5
A0A0D2XQW0
RKK80373.1
I1RWN2
b
Mock
A0A0M9EVJ7
A0A0D2XZ19
Col-0
mik2-1
c
ROS production (RLU min-1)
Col-0
mik2-1
bak1-5
Time (min)
d
ROS production (RLU min-1)
Col-0
mik2-1
bak1-5
Time (min)
e
DNA topoisomerase_A0A0M9EVJ7
547
DTGPSTSGRGRGR
560
PF01751
PF01131
668
Senescence domain-containing protein_A0A0D2XZ19
PF06911
512
180 YVQGSHSSHTGGR 193
f
Magnaporthe spp.
Neurospora spp.
Trichoderma spp.
Fusarium spp.
Verticillium spp.
Botrytis spp.
Time (MYA)
0
100
200
300
394

Supplementary Figure 11. Identification and characterization of active SCOOP-like peptides from *Fusarium* proteomes

(**a**) Fresh weight of 14-day-old seedlings grown in the presence of *Fusarium*-derived peptides at 10 µM concentration for 10 days. Line represents mean; error bars represent S.D. Dashed red line indicates the mock mean value. (F9G0P6 and F9F73C *n*=8 seedligns ; all other peptides *n*=12 seedlings) (**b**) Images of 14-day-old seedlings grown in the presence of *Fusarium*-derived peptides at 10 µM concentration for 10 days. (**c** and **d**) ROS production in leaf discs collected from 4-week-old *A. thaliana* plants induced by (**c**) 1 µM A0A0M9EVJ7 or (**d**) 1 µM A0A0D2XZ191 application (*n*=8 leaf disks). Points represent mean; error bars represent S.E.M. Integrated data shown in Figure 4 (d and e). (**e**) Schematic representation of the *Fusarium* proteins from which the active peptides were derived. Green areas indicate regions with homology to Pfam domains. Red areas indicate SCOOP-like motifs highlighted. Underlined amino acid positions indicate the amino acid at this position is not shared with any *Arabidopsis* SCOOPs. (**f**) Tree of predicted lineage divergence generated using TimeTree for the lineages shown in (e and f) with *Botrytis cinerea* included as an outgroup. (**a** and **b**) were performed only once as a screen, whilst (**c** and **d**) were performed three times with similar results.

Peptide	Sequence	Reference
flg22	QRLSTGSRINSAKDDAAGLQIA	51
Pep1	ATKVKAKQRGKEKVSSGRPGQHN	52
elf18	acetyl-MSKEKFERTKPHVNVGTI	53
SCOOP1	ETPPSRSRRGGGG	
SCOOP2	PVRSSRSPRSPSF	
SCOOP4	ASFHSASPKDKGP	
SCOOP5	IVRRSRSQRGRQY	
SCOOP6#1	IAGSSPSGQAPNI	
SCOOP6#2	EARPSKSKKGGGR	
SCOOP7	RAGPSKSGQGGGR	
SCOOP8	DFEGSISGQAGGG	
SCOOP9	GTGPSHSGHGGSS	
SCOOP10	FTGPSGSGHGGGR	
SCOOP11#1	GTPSSTSDRGGGG	
SCOOP11#2	DVGASSSGQGGGR	
SCOOP12	PVRSSQSSQAGGR	12
SCOOP13	YLPPSKSRKGKGP	
SCOOP14	FVPPSTSHKGQGP	
SCOOP12- Δ1	PVRSSQSSQAGG	

Supplementary Table 1. List of synthetic peptides used in this study.

Gene identifier	Peptide sequence	Proteome	Species
RKL12880.1	PVPSSQSSQSLPR	UP000286305	*Fusarium oxysporum*
RKK91479.1	PTRSSQSSQSNTA	UP000285237	*Fusarium oxysporum*
KIL85672.1	RIKSSQSSQAGIP	PRJNA253730	*Fusarium avenaceum*
A0A0D2XZ19	YVQGSHSSHTGGR	UP000009097	*Fusarium oxysporum* f. sp. *lycopersici*
I1RWN2	QLPSSRSGRGPGM	UP000070720	*Fusarium graminearum*
X0K0D8	LELQSRSARGGGT	UP000030685	*Fusarium oxysporum* f. sp. *cubense tropical race 4*
A0A0D2XQW0	PTPSSTSGKGGSA	UP000009097	*Fusarium oxysporum* f. sp. *lycopersici*
A0A0D2XVW5	VQTPSRSGDGGDV	UP000009097	*Fusarium oxysporum* f. sp. *lycopersici*
A0A098DTX8	RTAGSQSGHGGDE	UP000070720	*Fusarium graminearum*
A0A0M9EVJ7	DTGPSTSGRGRGR	UP000037904	*Fusarium langsethiae*
RKK80373.1	VAGPSRSGGQGGP	UP000285237	*Fusarium oxysporum* f. sp. *radicis-cucumerinum*
F9FC73	ELRRSTSSRSGGA	UP000002489	*Fusarium oxysporum (*strain *Fo5176)*
F9G0P6	KTPSSKSGRSDGS	UP000002489	*Fusarium oxysporum (*strain *Fo5176)*

Supplementary Table 2. SCOOP-like peptide sequences from *Fusarium* proteomes identified through BLAST and MAST analysis.

Use	Genotype/construct	Sequence
Genotyping	*mik2-1*	tttgactttgttcccagttgg
Genotyping		atgaaccgtttctctggaacc
Genotyping	*mik2-2*	caaagggaatagtttctccgg
Genotyping		tttgtagactttgccgtgtcc
Genotyping	*mik2-4*	cttgggtcaatccaaacacaagcagc
Genotyping		gctggttgcattcatcctctcaaac
Genotyping	*sobir1-13*	tagggcatacaatgctgaagc
Genotyping		tcaagaactaatgtggccagc
Genotyping	SALK left border	attttgccgatttcggaac
Genotyping	FLAG left border	cgtgtgccaggtgcccacggaatagt
Cloning	*MIK2:EFR*	cacaaggatcgaaacctagttgtcagtggtatttgtataggtatagc
Cloning		ccactgacaactaggtttcgatccttgtgtgatttctttgagg
Cloning	*EFR:MIK2*	gcctctgtcagttagaaagaaaatcatctacatactagttccgatcatcg
Cloning		cgatgatcggaactagtatgtagatgattttctttctaactgacagaggc
Cloning	*MIK2.1 cDNA*	caccatgaacaaaacaaacccag
Cloning		agaaaaggcagtggagatagagagc
Cloning	pMIK2::MIK2 gDNA	gtagagggtctccggaggcagtttgtatgattggtgccaa
Cloning		aatggcggtctcccgaagaaaaggcagtggagatagagagc
Cloning	M13	tgtaaaacgacggccagt
Cloning		caggaaacagctatgaccatg

Supplementary Table 3. Primers used in this study.

Name	Gene model
SCOOP1	AT5G44565.1
SCOOP2	AT5G44567.1
SCOOP4	AT5G44568.1
SCOOP5	AT5G44570.3
SCOOP6	AT5G44572.1
SCOOP7	AT5G44574.1
SCOOP8	AT5G44575.1
SCOOP9	AT5G44578.1
SCOOP10	AT5G44580.1
SCOOP11	AT5G44582.1
SCOOP12	AT5G44585.1
SCOOP13	AT1G22885.1
SCOOP14	AT1G22890.1

Supplementary Table 4. Gene models corresponding to SCOOP peptides studied.
