# Supplementary material for: Perception of a divergent family of phytocytokines by the Arabidopsis receptor kinase MIK2
Source: Nat Commun. 2021 Jan 29;12:705. doi: 10.1038/s41467-021-20932-y (PMC7846792; doi:10.1038/s41467-021-20932-y)
Supplement: Supplementary file 3 — Reporting Summary [file 41467_2021_20932_MOESM3_ESM.pdf]

nature research

Corresponding author(s): Prof. Cyril Zipfel

Last updated by author(s): Dec 21, 2020

Reporting Summary

Nature Research wishes to improve the reproducibility of the work that we publish. This form provides structure for consistency and transparency in reporting. For further information on Nature Research policies, see Authors & Referees and the Editorial Policy Checklist .

Statistics

For all statistical analyses, confirm that the following items are present in the figure legend, table legend, main text, or Methods section.

n/a	Confirmed	
☐	☒	The exact sample size (*n*) for each experimental group/condition, given as a discrete number and unit of measurement
☐	☒	A statement on whether measurements were taken from distinct samples or whether the same sample was measured repeatedly
☐	☒	The statistical test(s) used AND whether they are one- or two-sided<br>*Only common tests should be described solely by name; describe more complex techniques in the Methods section.*
☒	☐	A description of all covariates tested
☐	☒	A description of any assumptions or corrections, such as tests of normality and adjustment for multiple comparisons
☐	☒	A full description of the statistical parameters including central tendency (e.g. means) or other basic estimates (e.g. regression coefficient) AND variation (e.g. standard deviation) or associated estimates of uncertainty (e.g. confidence intervals)
☐	☒	For null hypothesis testing, the test statistic (e.g. *F*, *t*, *r*) with confidence intervals, effect sizes, degrees of freedom and *P* value noted<br>*Give P values as exact values whenever suitable.*
☒	☐	For Bayesian analysis, information on the choice of priors and Markov chain Monte Carlo settings
☒	☐	For hierarchical and complex designs, identification of the appropriate level for tests and full reporting of outcomes
☒	☐	Estimates of effect sizes (e.g. Cohen's *d*, Pearson's *r*), indicating how they were calculated

Our web collection on statistics for biologists contains articles on many of the points above.

Software and code

Policy information about availability of computer code

Data collection	Alignments and phylogenies: UGENE v.35.0<br>ROS data: Image32 (Photek, St Leonards on Sea, UK)<br>ITC: MicroCal PEAQ-ITC Analysis Software<br>Root skewing: ImageJ<br>Calcium data: SkanIt™ (Thermo Fisher Scientific)
Data analysis	Statistical analysis: GraphPad Prism v.7<br>Fungal species phylogeny: TimeTree (timetree.org)<br>Peptide similarity matices: SIAS (http://imed.med.ucm.es/Tools/sias.html)

For manuscripts utilizing custom algorithms or software that are central to the research but not yet described in published literature, software must be made available to editors/reviewers. We strongly encourage code deposition in a community repository (e.g. GitHub). See the Nature Research guidelines for submitting code & software for further information.

Data

Policy information about availability of data

All manuscripts must include a data availability statement. This statement should provide the following information, where applicable:

- Accession codes, unique identifiers, or web links for publicly available datasets
- A list of figures that have associated raw data
- A description of any restrictions on data availability

The authors declare that the data supporting the findings of this study are available within the paper and its supplementary information files.

Field-specific reporting

Please select the one below that is the best fit for your research. If you are not sure, read the appropriate sections before making your selection.

- [x] Life sciences
- [ ] Behavioural & social sciences
- [ ] Ecological, evolutionary & environmental sciences

For a reference copy of the document with all sections, see nature.com/documents/nr-reporting-summary-flat.pdf

Life sciences study design

All studies must disclose on these points even when the disclosure is negative.

Sample size	No sample size calculation was performed. Sample size was chosen as large as possible and in accordance with previous established protocols in the field. Given the sample size, adequate statistical analysis was performed.
Data exclusions	No data were excluded
Replication	All findings were successfully reproduced in several replicates.
Randomization	Due to the nature of the experimental setup randomizationwas not applicable
Blinding	Due to the nature of the experimental setup blinding was not applicable

Reporting for specific materials, systems and methods

We require information from authors about some types of materials, experimental systems and methods used in many studies. Here, indicate whether each material, system or method listed is relevant to your study. If you are not sure if a list item applies to your research, read the appropriate section before selecting a response.

Materials & experimental systems

n/a	Involved in the study	
[ ]	[x]	Antibodies
[ ]	[x]	Eukaryotic cell lines
[x]	[ ]	Palaeontology
[x]	[ ]	Animals and other organisms
[x]	[ ]	Human research participants
[x]	[ ]	Clinical data

Methods

n/a	Involved in the study	
[x]	[ ]	ChIP-seq
[x]	[ ]	Flow cytometry
[x]	[ ]	MRI-based neuroimaging

Antibodies

Antibodies used	Commercial antibodies used: α-GFP-HRP (B-2, sc-9996 HRP, Santa Cruz, 1:5000 dilution), α-RFP (ab34771, abcam), α-pMAPK (p44/42 MAPK (Erk1/2) (Cell Signalling technology). Non-conjugated primary antibodies were detected using α-rabbit IgG (whole molecule)–HRP (A0545, Sigma, dilution 1:10000)<br>Published antibodies: α-BAK1 (Roux et al 2011), α-pS612 (Perraki et al 2018)
Validation	Validation statements of commercial primary antibodies are available from manufacturers: α-GFP-HRP (https://datasheets.scbt.com/sc-9996.pdf); α-RFP (https://www.abcam.com/rfp-antibody-biotin-ab34771.html); α-pMAPK (p44/42 MAPK (Erk1/2) (https://www.cellsignal.co.uk/products/primary-antibodies/phospho-p44-42-mapk-erk1-2-thr202-tyr204-antibody/9101).

Eukaryotic cell lines

Policy information about cell lines

Cell line source(s)	Eukaryotic cell lines were solely used for the purpose of production of recombinant proteins.<br>Trichoplusia ni Tnao38 cells: Boyce Thompson Institute at Cornell University, Tower Road, Ithaca, NY, 14853, USA. (Ref.49)<br>Spodoptera frugiperda ,SF9: Thermo Fisher Scientific commercial cell line.
Authentication	Non of the cells used were authenticated
Mycoplasma contamination	The cells were not tested for mycoplasma contamination.

Commonly misidentified lines<br>(See ICLAC register)	not applicable
