# Supplementary material for: Perception of a divergent family of phytocytokines by the Arabidopsis receptor kinase MIK2
Source: Nat Commun. 2021 Jan 29;12:705. doi: 10.1038/s41467-021-20932-y (PMC7846792; doi:10.1038/s41467-021-20932-y)

Fig 1e

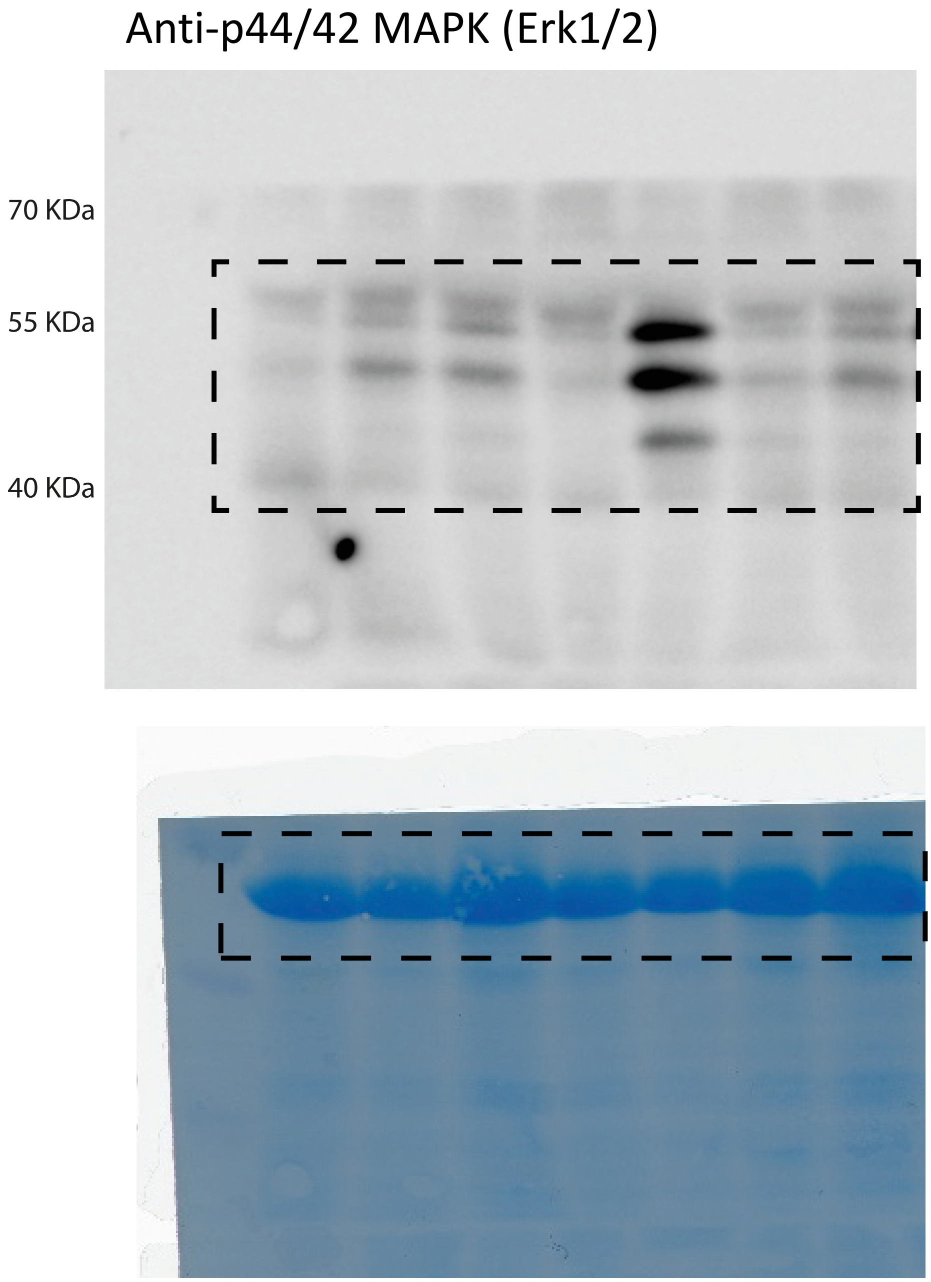

Fig 2a

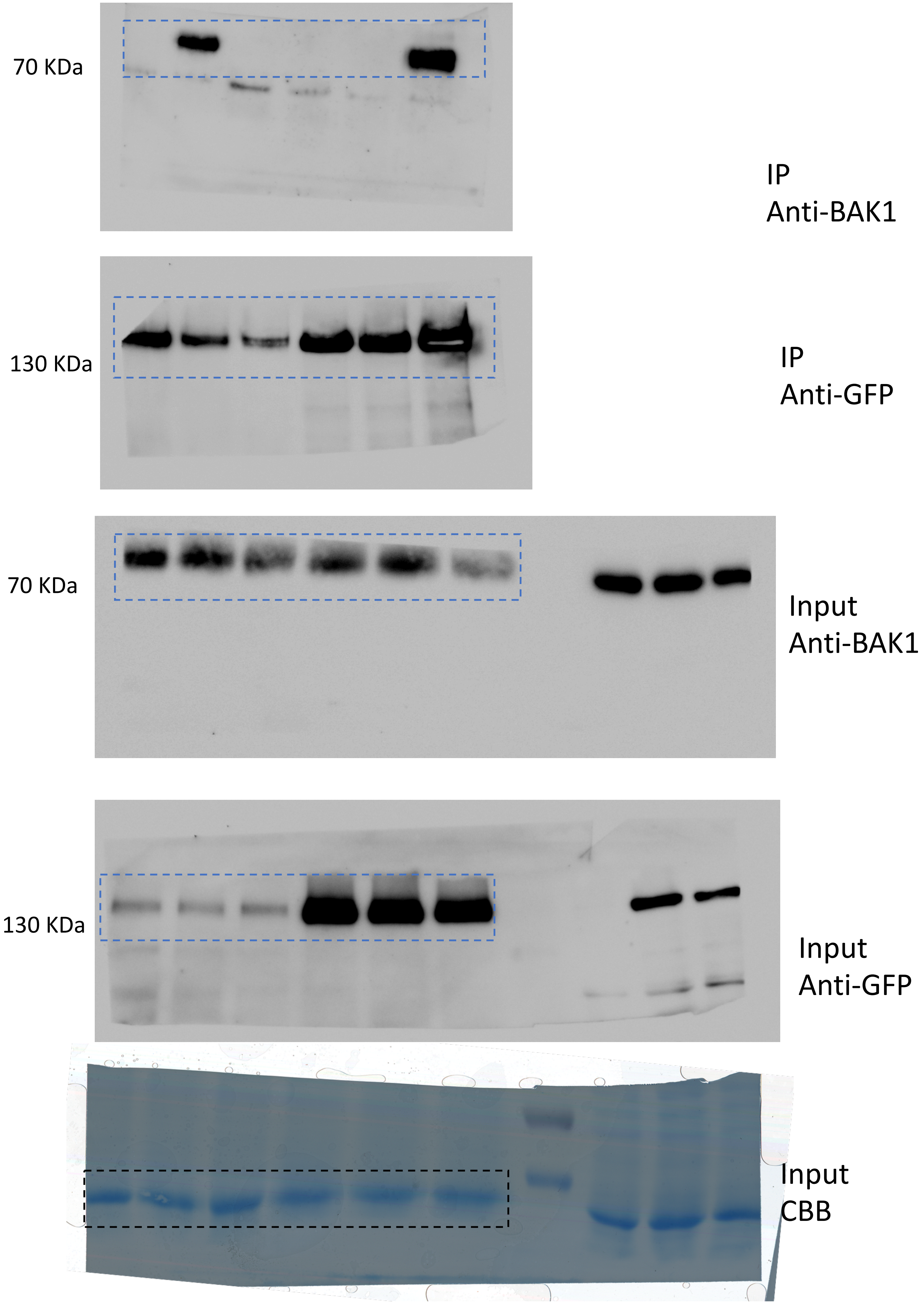
70 KDa
IP
Anti-BAK1
130 KDa
IP
Anti-GFP
70 KDa
Input
Anti-BAK1
130 KDa
Input
Anti-GFP
Input
CBB

Fig 2b

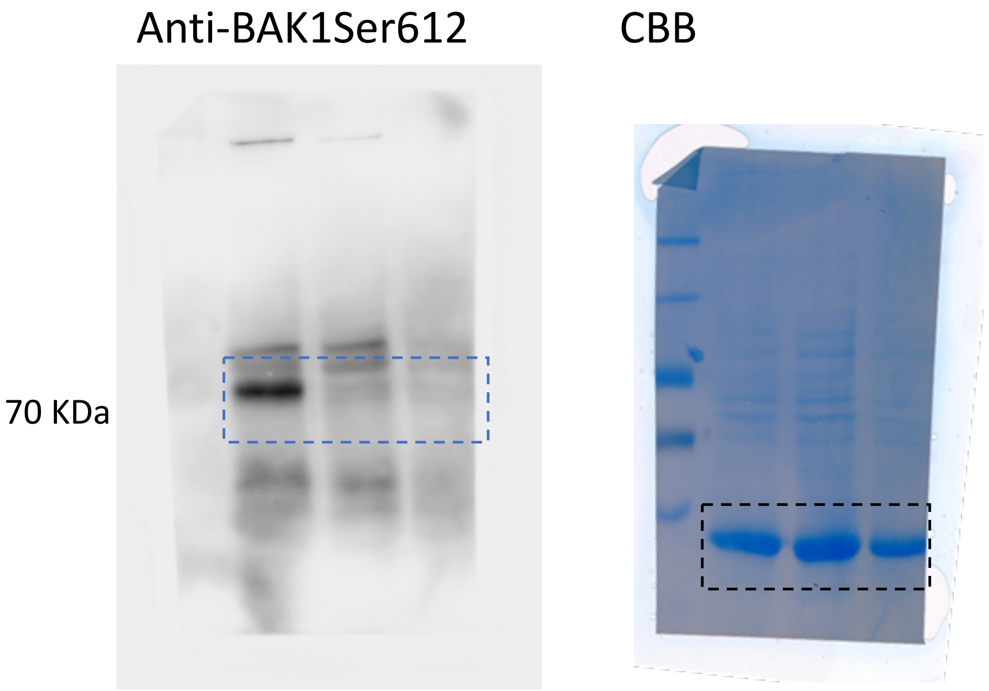

Fig 2e

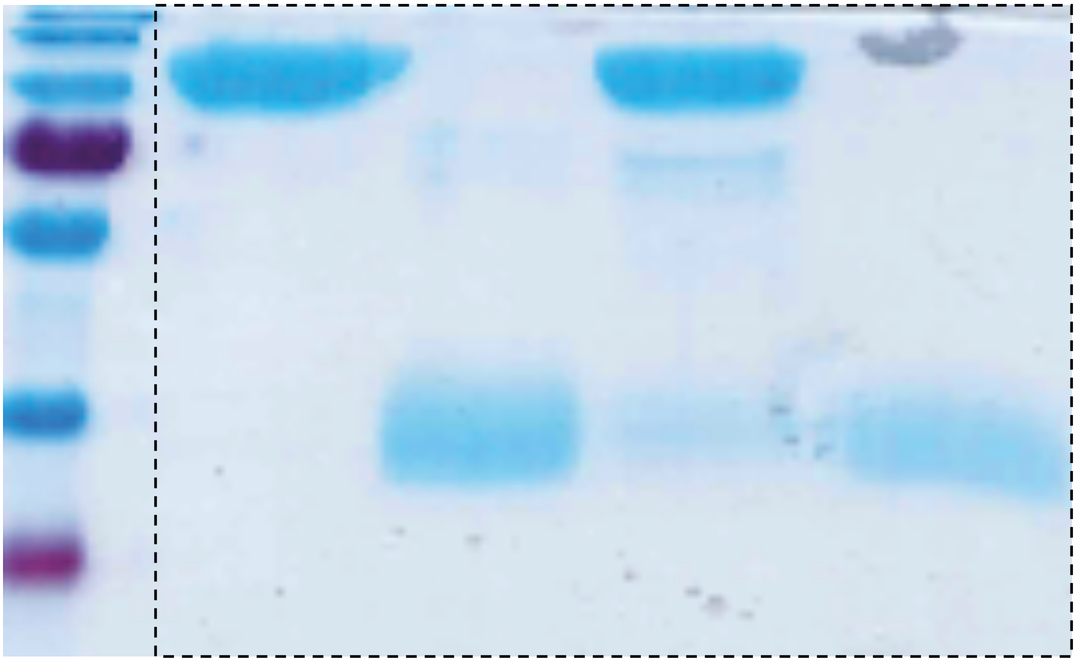

Fig 3d

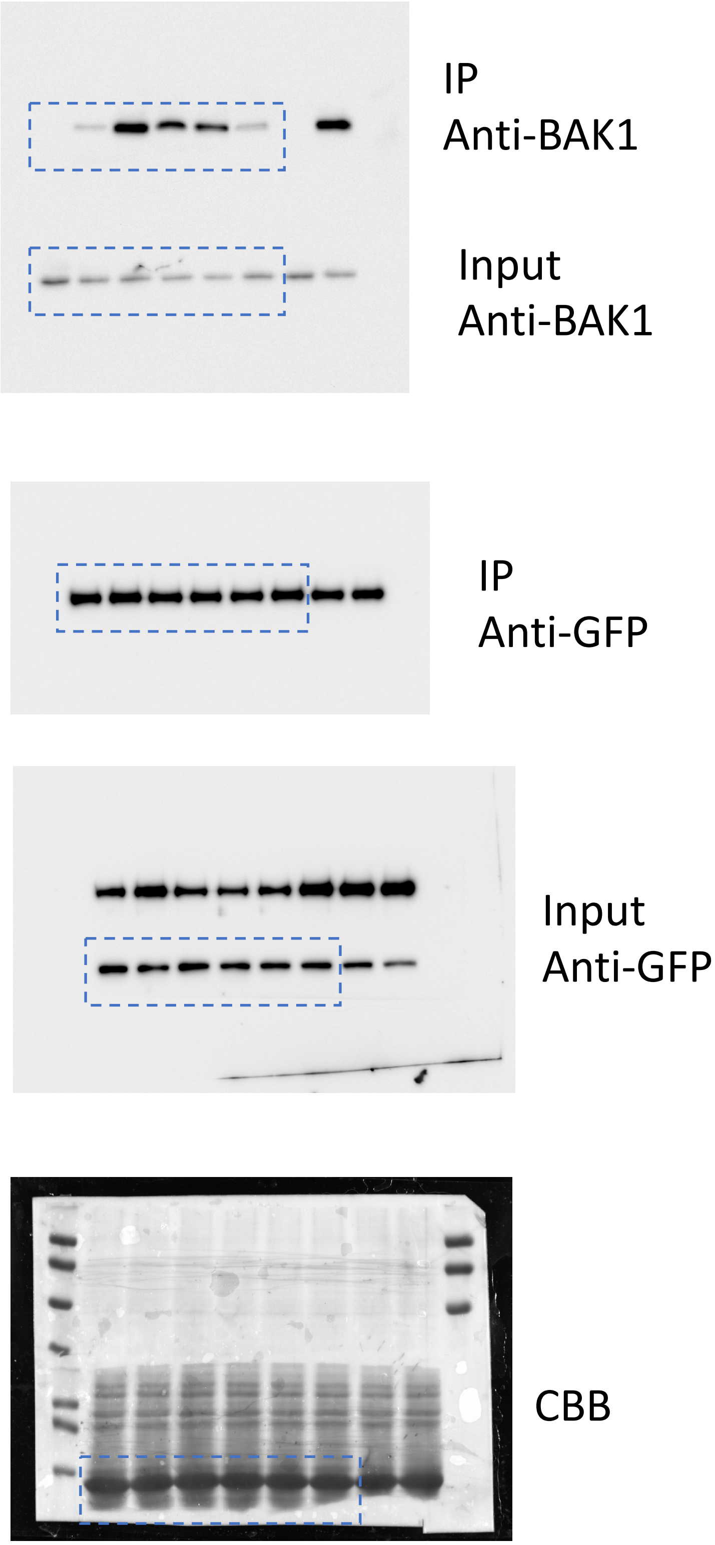

Figure 4c

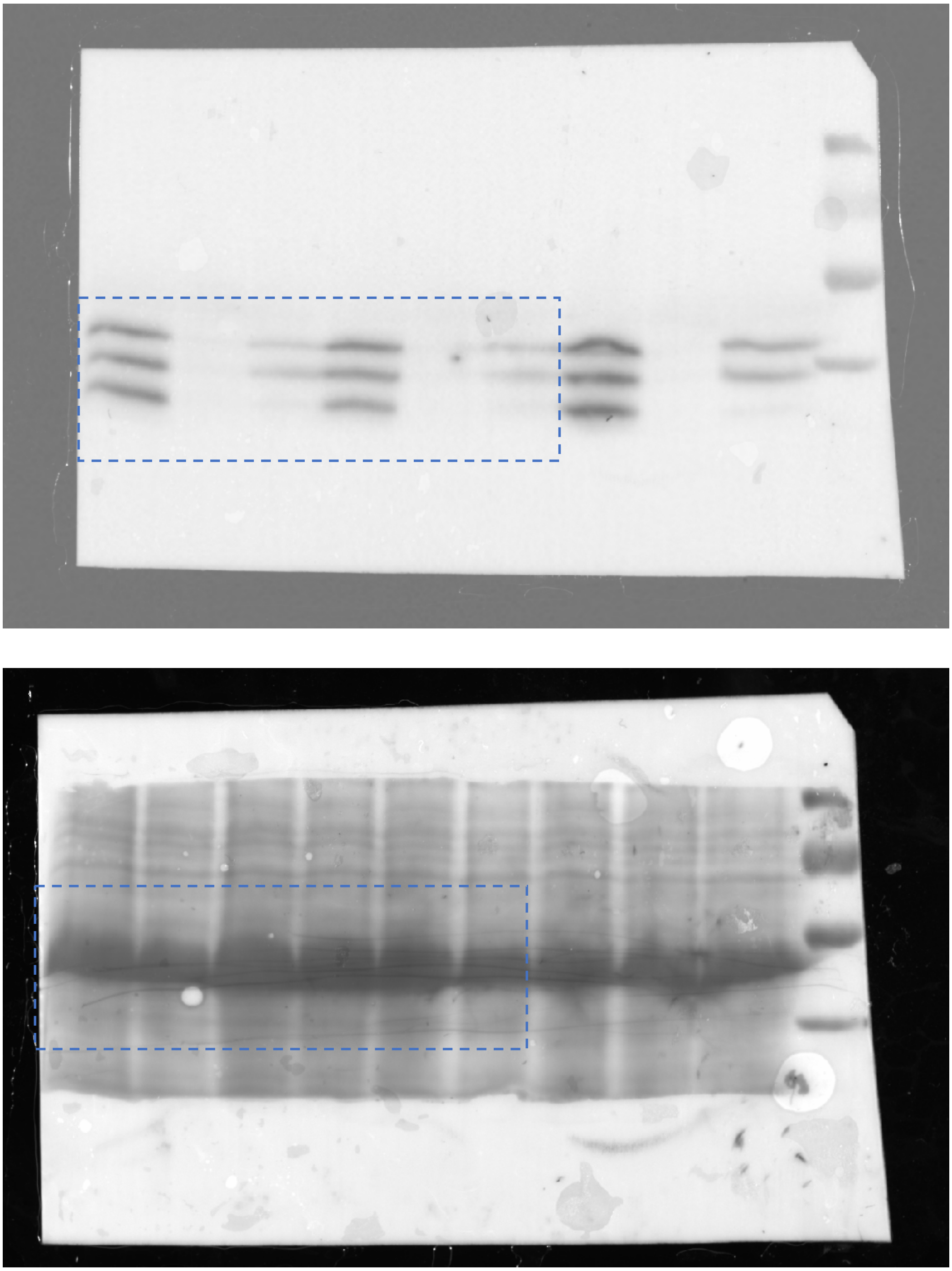

Figure 4f

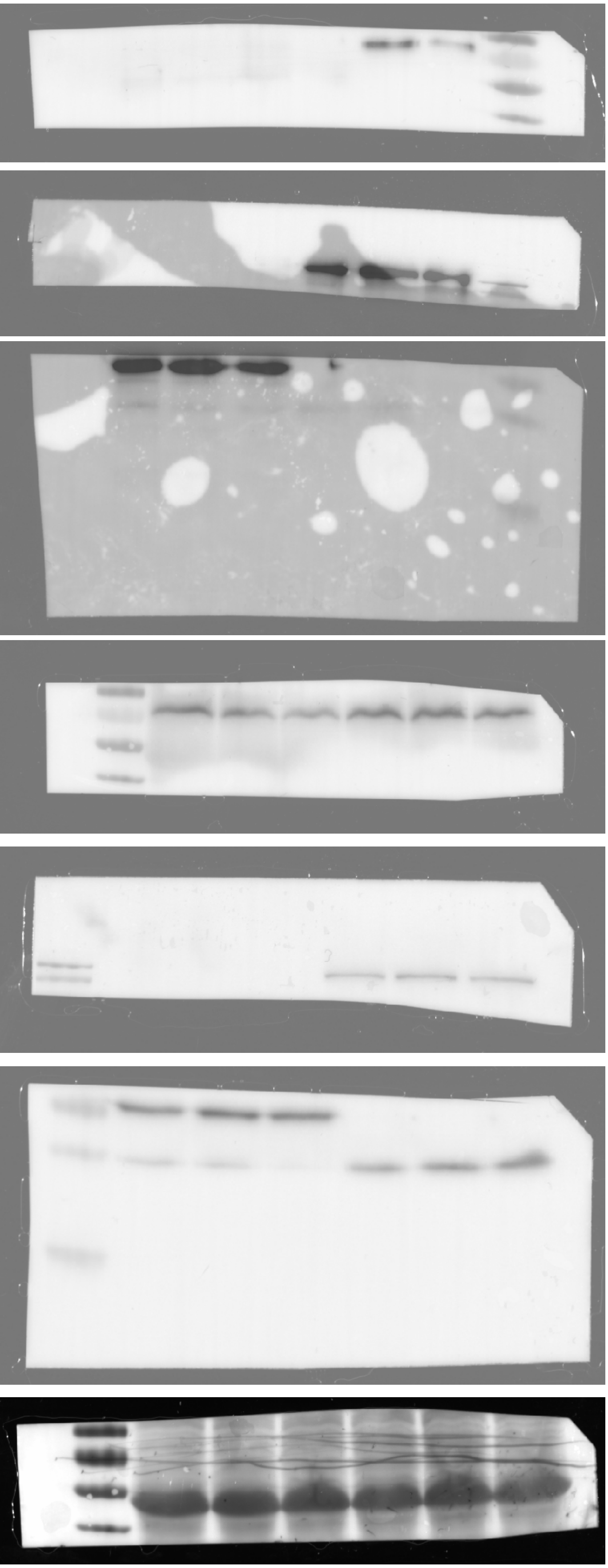

IP
Anti-BAK1

IP
Anti-GFP

IP
Anti-GFP

Input
Anti-BAK1

Input
Anti-GFP

Input
Anti-GFP

Input
CBB

Supplementary Figure 3c

70 KDa

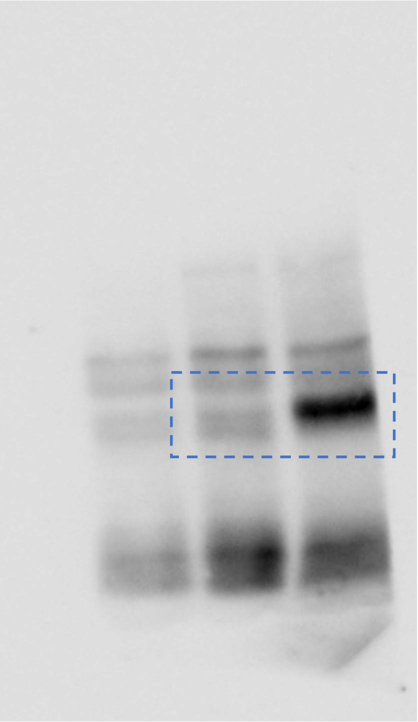

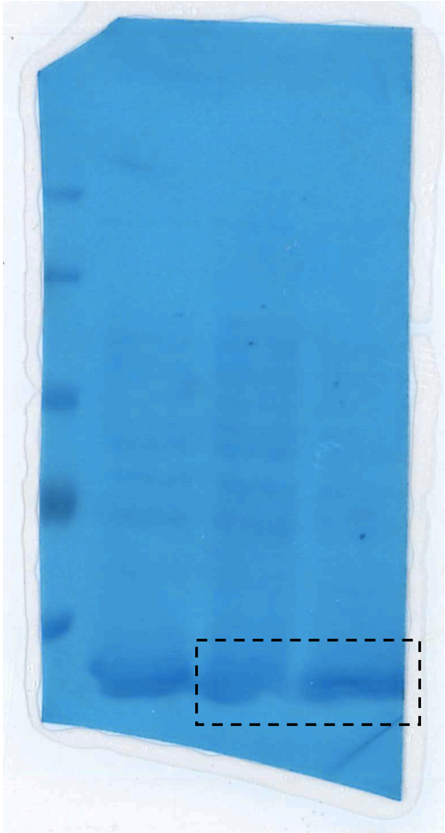

Supplementary Figure 5

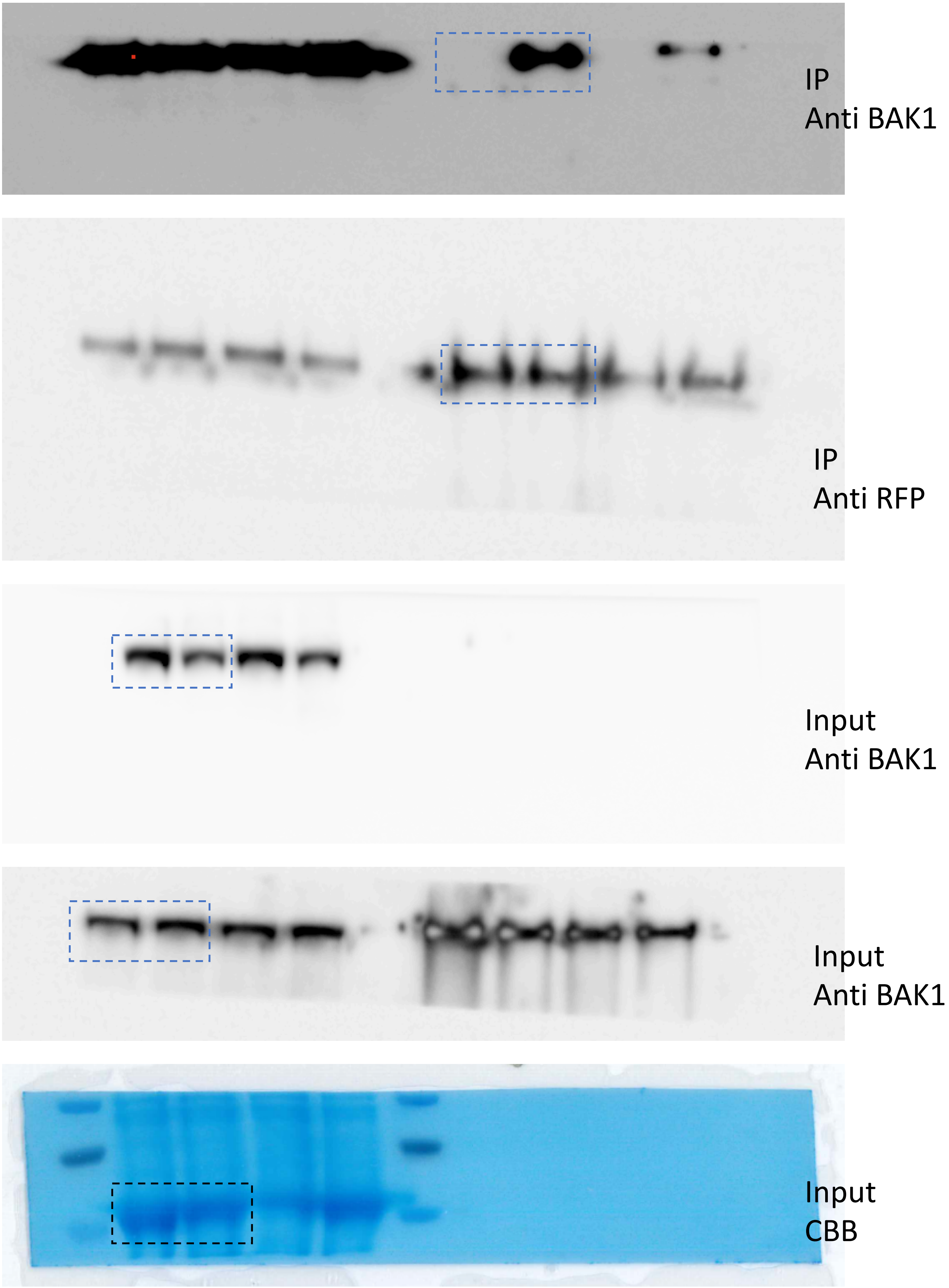

Supplementary Figure 4d

Anti-GFP

130 kDa

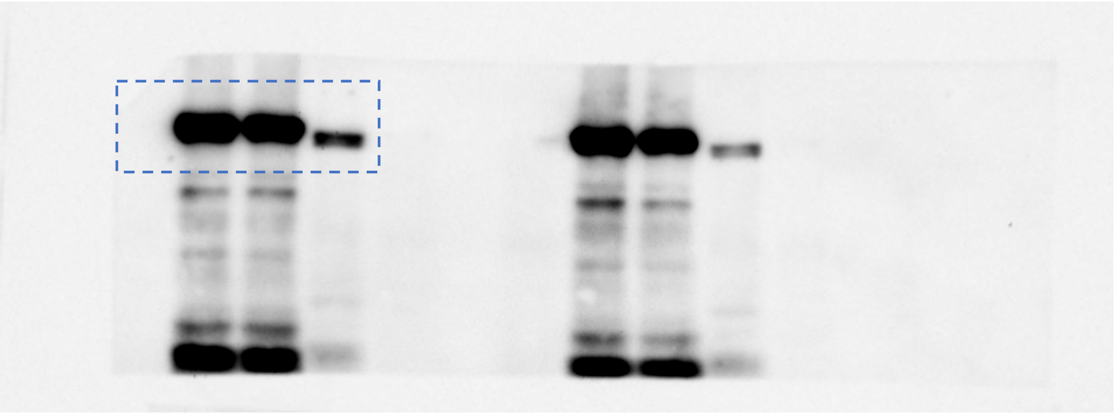

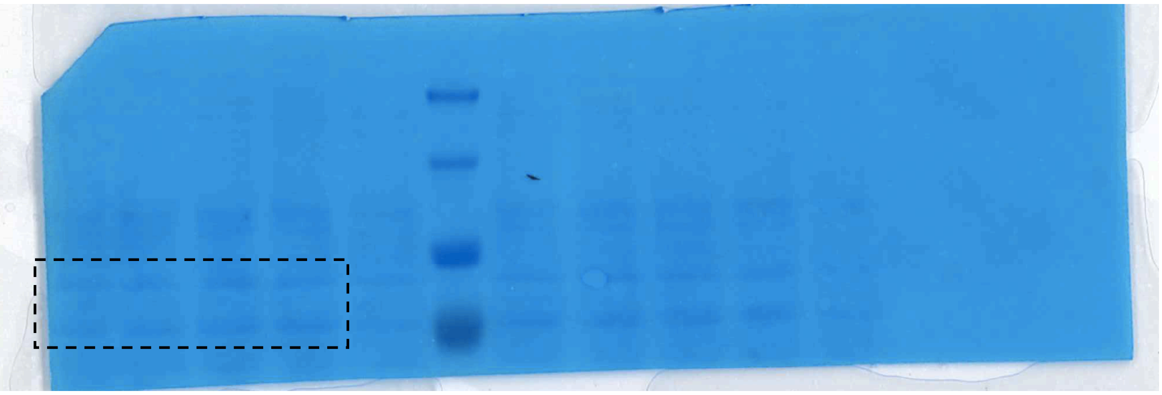

Supplement: Supplementary file 4 — Source Data [file 41467_2021_20932_MOESM4_ESM.zip › Rhodes_Source_Data/Rhodes_Source_data_full_blots.pdf]
