# Supplementary material for: Perception of a divergent family of phytocytokines by the Arabidopsis receptor kinase MIK2
Source: Nat Commun. 2021 Jan 29;12:705. doi: 10.1038/s41467-021-20932-y (PMC7846792; doi:10.1038/s41467-021-20932-y)
Supplement: Supplementary file 2 — Peer Review File [file 41467_2021_20932_MOESM2_ESM.pdf]

Editorial Note: This manuscript has been previously reviewed at another journal that is not operating a transparent peer review scheme. This document only contains reviewer comments and rebuttal letters for versions considered at *Nature Communications.*

REVIEWERS' COMMENTS

Reviewer #3 (Remarks to the Author):

The authors mostly responded positively to all the comments raised by three reviewers by providing additional data sets. In particular, they showed that the SCOOP peptide was effective even at nanomolar concentrations, which strongly indicates that this peptide is active at physiological concentrations. The authors also showed that the ability of distinct SCOOP peptides to induce the formation of a MIK2-BAK1 complex correlates well with their bioactivities. Although the mature structure of the SCOOP peptide in vivo has not been determined due to technical limitations, the conclusion is sufficiently validated by showing as much data as possible.
